# Air pollution modulates brown adipose tissue function through epigenetic regulation by HDAC9 and KDM2B

**DOI:** 10.1172/jci.insight.187023

**Published:** 2025-09-23

**Authors:** Rengasamy Palanivel, Jean-Eudes Dazard, Bongsoo Park, Sarah Costantino, Skanda T. Moorthy, Armando Vergara-Martel, Elaine Ann Cara, Jonnelle Edwards-Glenn, Shyam Biswal, Lung Chi Chen, Mukesh K. Jain, Francesco Paneni, Sanjay Rajagopalan

**Affiliations:** 1 Cardiovascular Research Institute, Department of Medicine, Case Western Reserve University, Cleveland, Ohio, USA.; 2 Translational Gerontology Branch, National Institute on Aging, NIH, Baltimore, Maryland, USA.; 3 Center for Translational and Experimental Cardiology (CTEC), Department of Cardiology, University Hospital Zurich and University of Zurich, Schlieren, Switzerland.; 4 University Heart Center, University Hospital Zurich, University of Zurich, Zurich, Switzerland.; 5 Department of Environmental Health and Engineering, Johns Hopkins University, Baltimore, Maryland, USA.; 6 Department of Environmental Medicine, New York University School of Medicine, New York, New York, USA.; 7 Division of Biology and Medicine, Brown University, Providence, Rhode Island, USA.; 8 Harrington Heart and Vascular Institute, University Hospitals, Cleveland, Ohio, USA.

**Keywords:** Cell biology, Metabolism, Adipose tissue, Epigenetics, Glucose metabolism

## Abstract

Recent experimental and epidemiologic data have strongly associated air pollution in the pathogenesis of insulin resistance and type 2 diabetes mellitus. We explored the effect of inhalational exposure to concentrated ambient particulate matter smaller than 2.5 μm (PM_2.5_), or filtered air, using a whole-body inhalation system (6 hours/day, 5 days/week) for 24 weeks on metabolism and brown adipose tissue (BAT) function. Mechanistic evaluation of insulin resistance, glucose uptake with ^18^F-fluorodeoxyglucose positron emission tomography, alongside evaluation for differentially methylated regions, chromatin accessibility, and differential expression of genes was performed. PM_2.5_ exposure impaired metabolism through changes in key BAT transcriptional programs involved in redox stress, lipid deposition, fibrosis, and altered thermogenesis. Significant differential methylation and widespread chromatin remodeling was noted in BAT with PM_2.5_. Integrated analysis uncovered a role for the histone deacetylase HDAC9 and histone demethylase KDM2B. The latter demethylates Lys-4 and Lys-36 of histone H3. Specifically, studies using ChIP combined with quantitative PCR confirmed HDAC9 and KDM2B occupancy and reduced H3K36me2 on the promoter of target BAT genes in PM_2.5_ mice, while *Hdac9/Kdm2b* knockdown and overexpression increased and reduced BAT metabolism, respectively. Collectively, our results provide insights into air pollution exposure and changes in BAT and metabolism.

## Abstract 2

Particulate matter (PM_2.5_) induces insulin resistance by upregulating Hdac9 and Kdm2b, which inhibit brown adipose tissue thermogenesis and antioxidant activity, linking air pollution to metabolic dysfunction.

## Introduction

Compelling epidemiological links and experimental evidence have causally linked exposure to air pollution with the pathogenesis of insulin resistance and development of type 2 diabetes mellitus (T2D) (1). Recent evidence suggests that as much as 20% of the global burden of T2D mortality may be accounted for by exposure to air pollution consisting of fine particulate matter smaller than 2.5 μm (PM_2.5_) (2). In prior studies, the effects of inhaled PM_2.5_ in inducing a range of abnormalities important for the pathogenesis of insulin resistance, including oxidative stress, skeletal muscle insulin resistance, hepatic insulin resistance, endoplasmic reticulum (ER) stress, hypothalamic inflammation, and circadian dysregulation have been demonstrated (3–8). These mechanisms are ultimately thought to converge in abnormal whole-body metabolism and phenotypic manifestations consistent with T2D (9–12). The range of phenotypic manifestations in T2D has increased the appreciation of the potential dysregulation of epigenetic pathways responsible for transcriptional control of key metabolic cascades, which may ultimately converge in insulin resistance and abnormal whole-body metabolism (3, 13–16). Environmental triggers and chemicals have been shown to be facile mediators of epigenetic changes such as DNA methylation and histone modification, which play an essential role in controlling chromatin compaction state and DNA repair (17, 18). In this context, we have previously demonstrated the involvement of histone deacetylases 2, 3, and 4 (HDAC2, -3, and -4) and other epigenetic pathways in the development of insulin resistance and circadian disruption in mice exposed to air pollution (14). Brown adipose tissue (BAT) has recently been shown to play a central role in whole-body metabolism, through its function in the physiological turnover of endogenous metabolites such as glucose and fatty acid clearance, and as a major site for thermogenic disposition of caloric load and susceptibility to insulin resistance (19). In previous research, we demonstrated a significant impact of chronic inhalational exposure to concentrated ambient PM_2.5_ on BAT dysfunction and circadian disruption in tissues such as the liver (14, 20, 21). In this study, we investigated whole-body metabolism, genome-wide DNA methylation patterns, differential chromatin accessibility, and transcriptional and functional changes in BAT in response to air pollution exposure in order to uncover broad integrated regulators of metabolism and BAT function in response to air pollution.

## Results

### PM_2.5_ causes impaired glucose uptake and altered mitochondrial structure in BAT.

**Figure 1:**
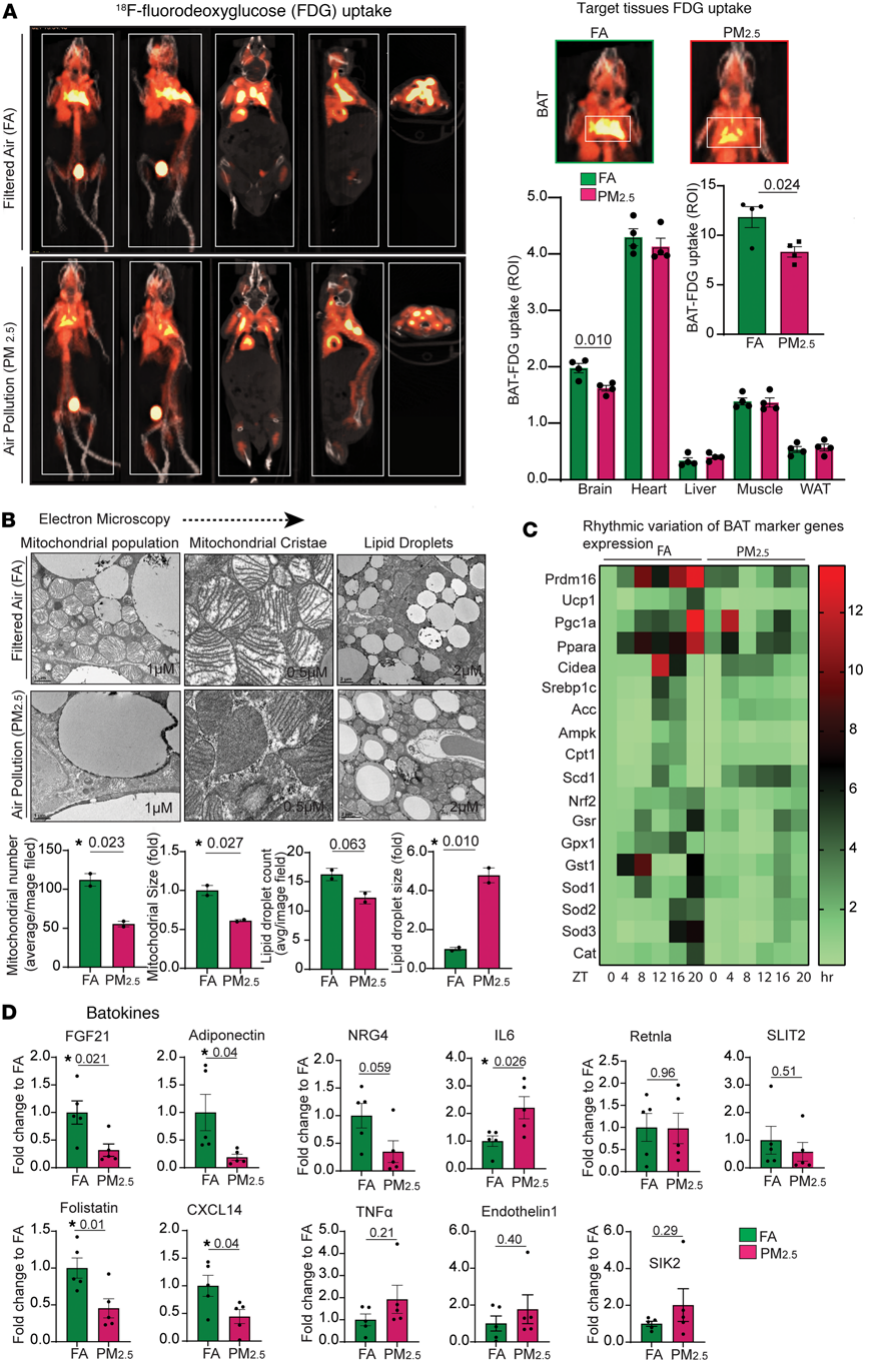
Impaired glucose uptake and altered ultrastructure and secretory function of BAT induced by air pollution. (**A**) FDG distribution in various peripheral tissues induced by insulin. Representative axial, coronal, sagittal images of mice from FA versus PM_2.5_ exposure (*n* = 4) are shown. PET/CT showing specific BATs that were assessed in this study (ROI placement in BAT in axial, coronal, and sagittal PET images and overlaid on CT images), and localization of specific tissues was established utilizing CT scans. Bar plots indicate mean FDG uptake level of BAT and other metabolic organs from mice exposed to FA versus PM_2.5_ (*n* = 4). (**B**) Representative TEM photomicrographs acquired from the section of the BAT from mice exposed to FA and PM_2.5_ for 24 weeks (*n* = 2). Bar plots represent mean mitochondrial number and size per image field. Higher magnification (scale bars: 0.5 μm) of mitochondria shows lamellar cristae in FA-exposed and tubular cristae structure in PM_2.5_-exposed mice. Lower magnification (scale bars: 2 μm) micrographs demonstrate the accumulation of lipid droplets in mitochondria. Bar plots represent the mean number of lipid droplets and their size per image field. Data were collected across 48 fields of view for 2 mice per group. (**C**) Heatmap indicates 24-hour circadian variation (ZT0 to ZT20) of thermogenic, metabolic, and antioxidant gene expression in BAT tissues (*n* = 3). Data are presented as fold change relative to the baseline (FA at ZT0) and the heatmap shows the mean value at each time point. Statistical significance was determined using an unpaired, 2-tailed Student’s *t* test, with *P* < 0.05 considered significant when comparing each ZT point between groups. (**D**) Describes the batokine mRNA expression levels in BAT from mice exposed to FA versus PM_2.5_ for 24 weeks (*n* = 5). Data are provided as mean ± SEM. *P < 0.05 versus FA-exposed mice by unpaired, 2-tailed Student’s *t* test.

Figure 1A depicts representative positron emission tomography (PET) images of the concentrated ambient particulate air pollution exposure group (henceforth referred to as PM_2.5_), compared with the filtered (FA) air controls. In BAT, the standard uptake value (SUV) of PM_2.5_-exposed mice was significantly lower than in FA-exposed mice (8.32 ± 0.52 vs. 11.83 ± 1.05; *P* < 0.02). In brain and heart, ^18^F-fluorodeoxyglucose (FDG) uptake showed a trend to lower levels in PM_2.5_-exposed mice, while uptake in liver, skeletal muscle, and white adipose tissue (WAT) was found to be comparable to control mice (Figure 1A). BAT in PM_2.5_-exposed mice exhibited dysmorphic mitochondria, with significant subcellular heterogeneity by transmission electron microscopy (TEM) (Figure 1B). Lower numbers of mitochondria were noticed in BAT of PM_2.5_-exposed mice, compared with FA-exposed mice. Strikingly, BAT of PM_2.5_-exposed mice exhibited abnormal lipid deposition, with large lipid droplet size and with comparable numbers of lipid droplets between groups (Figure 1B).

### PM_2.5_ alters rhythmicity of genes involved in circadian rhythm, mitochondrial biogenesis, and antioxidant response in BAT.

Decreased rhythmic expression of *Prdm16* mRNA was observed at zeitgeber time 8 (ZT8), ZT12, and ZT20 (*P* < 0.05) in PM_2.5_-exposed mice, compared with FA, encoding a key BAT transcription factor that regulates thermogenic gene programs in brown adipocytes (Figure 1C). Our data confirmed an approximately 3- to 4-fold decrease in *Prdm16* mRNA amount over a 24-hour period in PM_2.5_-exposed mice, compared with that of FA-exposed mice. Similarly, *Ucp1*, which exhibited a strong circadian rhythmicity with a peak at ZT12, was abrogated in PM_2.5_-exposed mice (Figure 1C). *Pgc1a*, *Ampk*, *Cpt1*, and *Cidea* by quantitative reverse transcription PCR (qRT-PCR) also displayed alternate patterns of rhythmicity (Figure 1C). Marked variations in temporal expression of key BAT antioxidant pathways, including *Nrf2* (encoding nuclear factor erythroid 2–related factor 2) and *GSH*-related genes (*GSR*, glutathione [*GSH*] system) (Figure 1C). In addition, we applied Cosinor analysis (Supplemental Figure 1; supplemental material available online with this article; https://doi.org/10.1172/jci.insight.187023DS1) to investigate the rhythmic variation of the genes noticed in Figure 1C. Compared with FA-exposed mice, PM_2.5_ exposure altered the 24-hour oscillations of metabolic and antioxidant genes in BAT. These included changes in the rhythm-adjusted mean (mesor) of *Prdm16*, *Cidea*, *Ampk*, *Cpt1*, *Ucp1*, *Nrf2*, *Cat*, *Sod2*, and *Sod3* (all reduced, *P* < 0.05), and *Scd1* (elevated, *P* < 0.05); changes in amplitude (the mean of the rhythm from peak to trough) of *Prdm16*, *Cidea*, *Srebp1c*, *Acc*, *Ampk*, and *Cpt1* (all reduced, *P* < 0.05), and *Sod3* (elevated, *P* < 0.05); as well as a changes in the acrophase (the hour at which the rhythm peaks) of *Pgc1a*, *Cidea*, *Srebp1c*, *Ampk*, *Gst1a*, and *Cat* (all *P* < 0.05) (Supplemental Figure 1).

### PM_2.5_ alters the “batokine” profile and induces inflammation and fibrosis in BAT.

The mRNA levels of *Fgf21*, *Adipoq*, *Fst*, *Cxcl14*, and *Nrg4* were strongly decreased (Figure 1D) in response to PM_2.5_. In contrast, *Tnfa* and *Il6* were higher in PM_2.5_ (Figure 1D). BAT in PM_2.5_-exposed mice had more extracellular matrix deposition (Supplemental Figure 2, A–C). *Gtf2ird1*, encoding a cold-inducible transcription factor that represses adipose tissue fibrosis through a PRDM16-EHMT1 complex, was decreased in PM_2.5_ mice (Supplemental Figure 2D). *Col5a1*, which encodes α1 (V) collagen, was upregulated in the BAT of PM_2.5_-exposed mice, with no changes in *Col1a* and *Col3a* expression (Supplemental Figure 2D). Apart from the above classical fibrotic markers, the transcription levels of other notable fibrotic markers such as *Angptl2*, *Mrtfa*, and *Mrtfb* were significantly and moderately upregulated by PM_2.5_ (Supplemental Figure 2D). The mRNA levels of fibrotic suppressor *Ehmt1* (encoding euchromatic histone lysine methyltransferase) were downregulated by PM_2.5_.

### PM_2.5_ causes impaired glucose homeostasis and energy expenditure.

**Figure 2:**
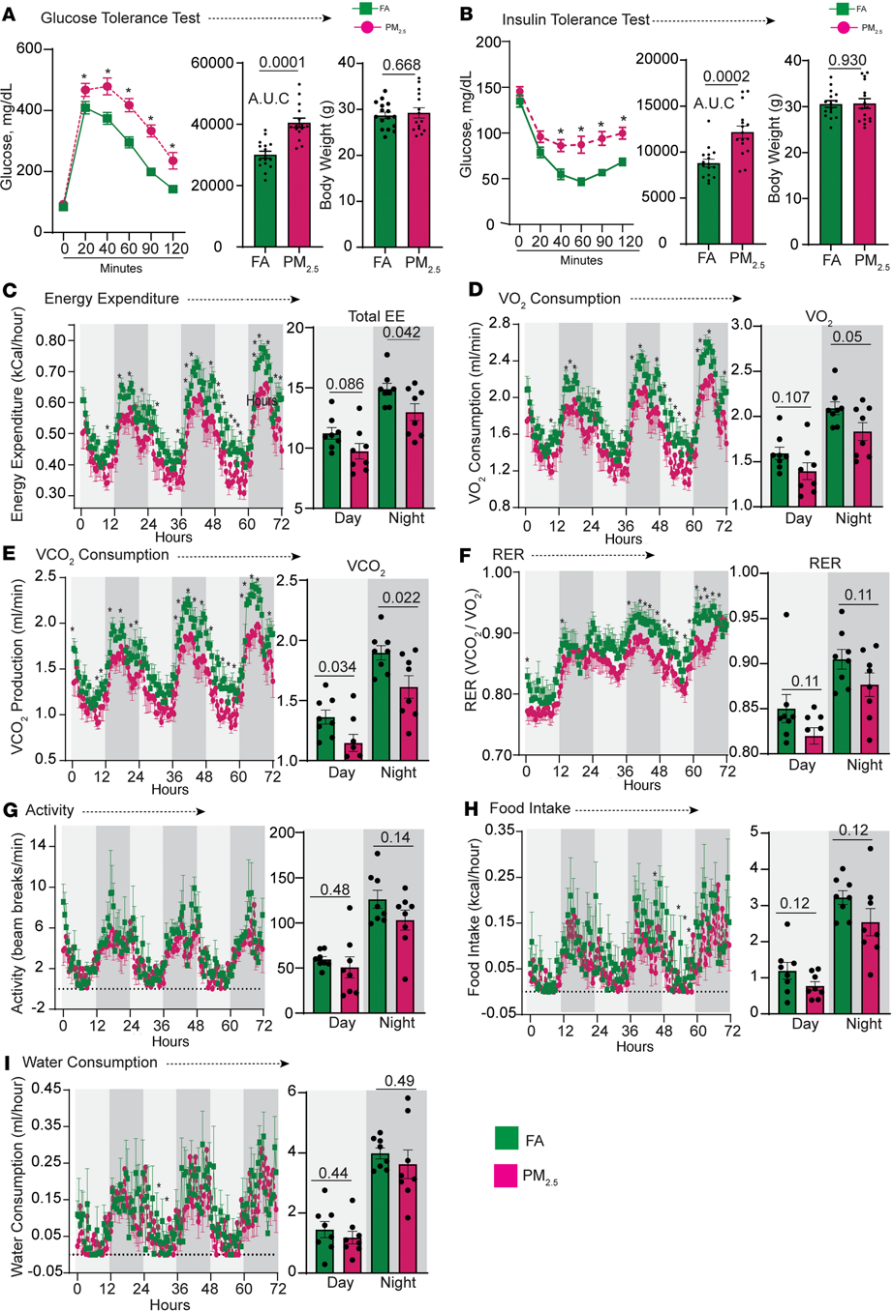
Impaired glucose clearance and metabolic rate induced by air pollution. (**A**) Glucose tolerance tests in FA- versus PM_2.5_-exposed mice (*n* = 16/group). After an overnight fast, an i.p. glucose load (2 g/kg) was given to FA- and PM_2.5_-exposed mice. (**B**) Insulin tolerance tests were done on 6-hour-fasted mice using an i.p. injection of 0.75 U/kg regular human insulin and blood glucose levels were monitored as indicated in the figures. The AUC of glucose and insulin tolerance test results and corresponding average body weight are shown in the bar plots. Energy expenditure, respiratory exchange ratio (RER), and physical activity were measured by indirect calorimetry in C57BL/6J mice after 20 weeks of exposure to FA or PM_2.5_. (**C**) Energy expenditure was calculated from measured VO_2_ and RER. It is shown respective to body weight for each exposure group as an average over a 72-hour period. Day period is represented by white background and night period by gray background. The corresponding bar plots indicate average total day and night energy expenditure. VO_2_ (**D**), VCO_2_ (**E**), RER (**F**), and physical parameters such as, activity level (**G**), food and water intake (**H**), and water consumption (**I**) are shown as average over a 72-hour period. Corresponding bar plots show average day and night total VO_2_, VCO_2_, and RER, activity, food and water intake values of the FA and PM_2.5_ groups. Metabolic cage study, all parameters, *n* = 8/group. Data are provided as mean ± SEM. *P < 0.05 versus FA-exposed mice by unpaired, 2-tailed Student’s *t* test.

We identified a marked and significant decrease in glucose and insulin clearance in PM_2.5_ compared with FA animals (Figure 2, A and B). PM_2.5_-exposed mice showed a significant reduction in energy expenditure during dark-phase conditions (Figure 2C). Oxygen consumption (VO_2_) and carbon dioxide production (VCO_2_) in PM_2.5_ mice were lower than in FA during both light and dark phases (Figure 2, D and E). The respiratory exchange ratio (RER) was not significantly different between groups during the light and dark phases (Figure 2F). However, during the light and dark phases, PM_2.5_-exposed mice showed a general decrease in RER, indicating a shift in substrate utilization toward fat oxidation (Figure 2F) (22). No difference in locomotor or ambulatory activity, or caloric or water intake was observed (Figure 2, G–I).

### Air pollution–induced DNA methylation patterns in BAT.

**Figure 3:**
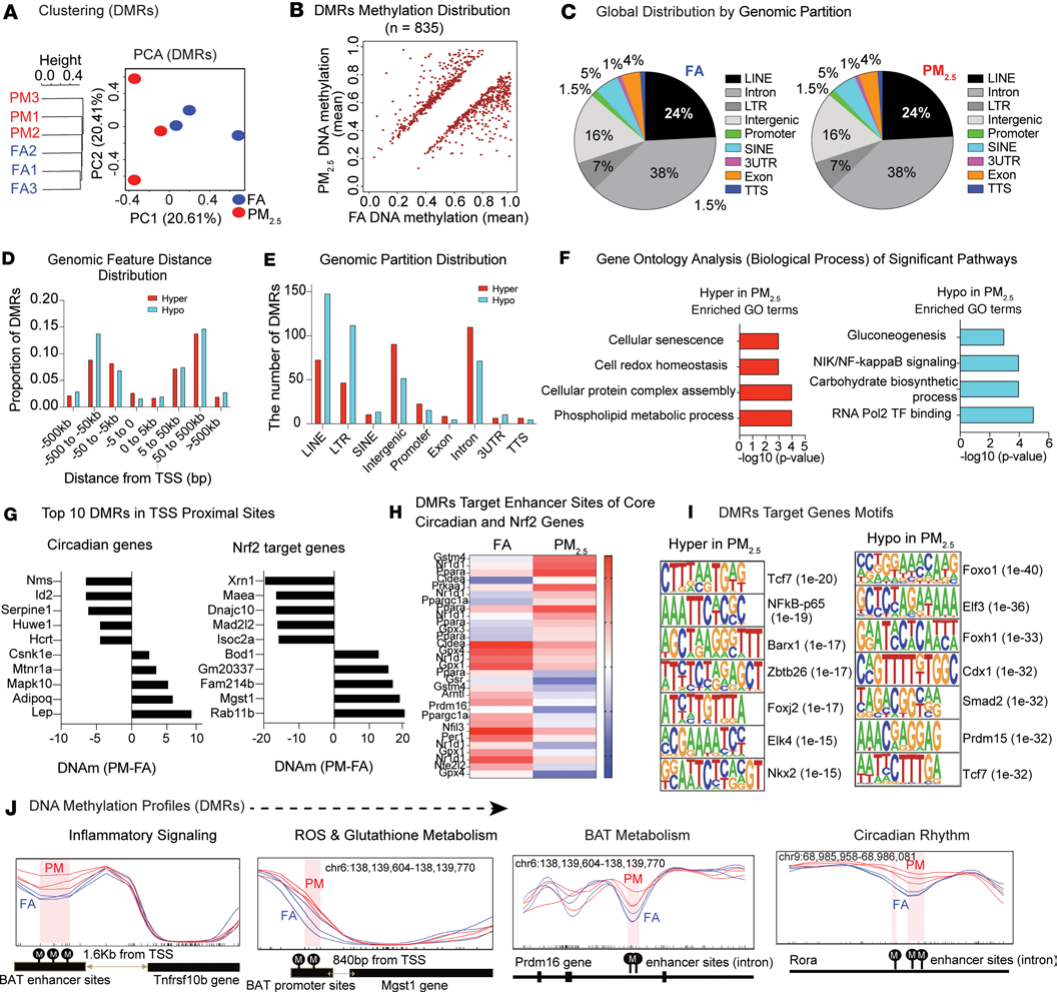
Methylome data analysis of PM_2.5_-exposed BAT. (**A**) Hierarchical clustering dendrogram and PCA plot of biological replicates from FA (*n* = 3) and PM_2.5_ (*n* = 3) in the methylome data of BAT. (**B**) Scatter plot of DNA methylation levels of statistically significant differentially methylated regions (DMRs: 881, of which 464 are hypomethylated and 417 hypermethylated) between FA- and PM_2.5_-exposed BAT. The *x* axis is the mean FA DNA methylation level (0%–100%) and the *y* axis is the mean PM_2.5_ DNA methylation level (0%–100%). (**C**) Pie charts (%) of global distribution of all CpG sites in predefined genomic partitions by PM exposure group. Left: FA samples. Right: PM_2.5_ samples (binned 100 bp). (**D** and **E**) Bar plots of GREAT analysis in PM_2.5_-exposed BAT.(**D**) Genomic feature distance (DMRs to TSSs) distribution and (**E**) genomic partition distribution of DMRs. (**F**) GO analysis of DMR significant terms (Biological Process) by DNA methylation status. Left: Hypermethylated genes. Right: Hypomethylated genes. (**G**) DNA methylation levels in circadian rhythm and NRF2 pathway target genes. (**H**) Heatmap plot of significance of DMR enhancer and regulatory sites predicted by GREAT analysis for the core genes associated with circadian rhythm and NRF2 pathways. (**I**) Predicted DMR target motifs and regulatory enhancer sites by GREAT analysis. Comparison of significant transcription factor motifs of DMR target genes by DNA methylation status. Left: Hypermethylated DMR target genes. Right: Hypomethylated DMR target genes. (**J**) Illustration of CpG differential methylation profiles of DMRs in distal or proximal target sites of specific genes and pathways of interest. The *y* axis shows the DNA methylation level (0%–100%) with a smoothing line, and the *x* axis shows the corresponding CpG genomic location. Each *x*-axis tick denotes a CpG site. Circled “M” symbols denote DMR CpG sites. Far left: Distal enhancer site of *Tnfrsf10b* (Inflammatory Signaling). Middle left: Proximal promoter site of *Mgst1* (ROS and Glutathione Metabolism). Middle right: Promoter site of *Prdm16* (BAT Metabolism). Far right: Promoter site of *Rora* (Circadian Rhythm).

Figure 3A shows hierarchical clustering analysis and principal component analysis (PCA) of BAT for FA- and PM_2.5_-exposed mice in biological replicates. We identified a total of 881 differentially methylated regions (DMRs, see Methods). Out of these DMRs, 464 were hypomethylated (PM_2.5_ < FA) and 417 were hypermethylated (PM_2.5_ > FA). After further reducing the dimensionality of the data and selecting out missing or redundant gene annotations, a list of 441 unique fully annotated DMRs was assembled (234 hypomethylated and 237 hypermethylated, see Supplemental Table 1A). The distribution of DNA methylation levels of significant DMRs is also shown in Figure 3B. Figure 3C depicts the global proportions of identified methylated CpG sites per genomic partition (long interspersed nuclear element [LINE], long terminal repeat [LTR], short interspersed nuclear element [SINE], Intergenic, Promoter, Exon, Intron, 3′UTR, transcription start site [TTS]) by exposure groups. No global difference in the proportions of methylation per genomic partition between FA- and PM_2.5_-exposed mouse samples was detected. DMRs were primarily localized in LINE, Intron, LTRs, and Intergenic partitions, with the total number of DMRs much lower in Promoter, SINE, 3′UTR, Exon, and TSS partitions. When DMRs were categorized according to their distance from the TSS, we observed that DMRs were primarily localized around 50–500 kb upstream or downstream of the TSS (Figure 3D). We then examined the total number of identified DMRs according to hyper- or hypomethylation status by genomic partition (LINE, SINE, LTR, Intergenic, Intron, Exon, 3’UTR, Promoter, and TSS) in the FA and PM_2.5_ samples. Interestingly, there were more hypomethylated DMRs identified in the LINE and LTR partitions, while hypermethylated DMRs were more common in Intergenic, Intron Promoter, and Exon partitions (Figure 3E). Figure 3F depicts the results of significantly enriched Gene Ontology (GO) terms (in Biological Process) of DMRs according to DNA methylation status. Hypomethylated regions corresponded to gluconeogenesis, inflammation, and carbohydrate biosynthesis, while hypermethylated regions predicted pathways involving cellular senescence, redox stress, etc.

Given the findings of altered circadian function and redox stress, we explored potential methylation pathways that could explain circadian and *Nrf2* dysregulation, especially given the central role of NRF2 as a redox transcription factor (23). First, we identified the promoter sites within ±1 kb of the corresponding TSSs of circadian and NRF2 target genes. Second, we predicted the corresponding enhancer sites using published histone ChIP-seq datasets (enriched peaks from *H3K27ac* and *H3K4me1*). Figure 3G shows the DMRs in the promoter and enhancer regions of the top 5 and bottom 5 target genes associated with circadian rhythm and NRF2. Among circadian rhythm target genes, we found *Lep*, *Adipoq*, and *Mapk10* to be hypermethylated, while *Nms*, *Id2*, and *Serpine1* were hypomethylated in PM_2.5_-exposed mice. Figure 3H shows the corresponding heatmap of significant DMR enrichment levels in the enhancer regions of circadian and NRF2 target genes for the FA and PM_2.5_ sample groups. We also performed de novo motif prediction to identify potential transcription factor binding sites (TFBSs) that overlap with DMRs. Hypermethylated DMRs in the PM_2.5_ samples corresponded to sites enriched for the TFBSs of *Tcf7*, *Nfkb*-*p65*, and *Barx1*, while hypomethylated DMRs in the PM_2.5_ samples were found to be enriched in the TFBSs of *Foxo1*, *Elf3*, and *Foxh1* (Figure 3I). Figure 3J illustrates differential methylation profiles of DMRs in proximal (DMR-to-TSS < 2 kb) or distal (DMR-to-TSS > 2 kb) target sites of specific regulated genes and pathways involving in inflammatory ROS/glutathione metabolism, circadian rhythm, and BAT metabolism.

### Air pollution–induced DNA accessibility patterns in BAT.

**Figure 4:**
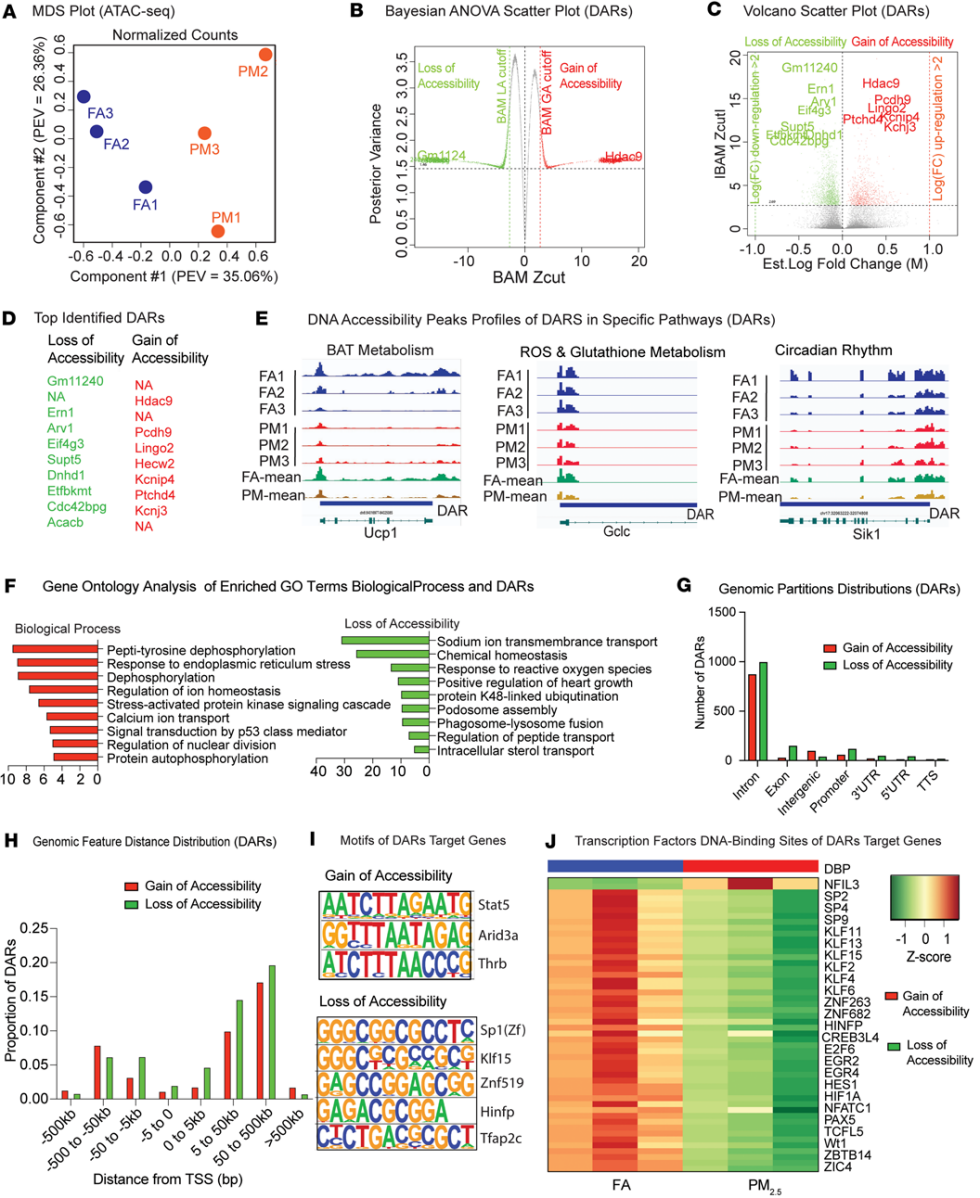
DNA accessibility data analysis of PM_2.5_-exposed BAT. (**A**) Multidimensional scaling plot of biological replicates from FA (*n* = 3) and PM_2.5_ (PM: *n* = 3) in the DNA accessibility data of BAT. (**B** and **C**) Bayesian ANOVA (BAM) “M” scatter plot of statistically significant differentially accessible regions (DARs: 2278, of which 833 are with a gain of accessibility [GA] and 1445 with a loss of accessibility [LA]) in PM_2.5_-exposed BAT. (**B**) The BAM “M” plot is a shrinkage plot, where each point represents a single DAR. Red and green dots indicate GA and LA DARs, respectively. The *y* axis is the posterior variance and the *x* axis is the Bayesian test statistic (Zcut) value. (**C**) The volcano plot, in which every point represents a single DAR, is a scatter plot of statistical significance versus magnitude-of-change, where the *y* axis represents the absolute value of the Bayesian test statistic (Zcut) and the *x* axis represents the log(fold change). (**D**) Table of top 10 significant DARs ordered by decreasing significance and by DNA accessibility status: GA (red) or LA (green). “NAs” refer to intergenic DARs peaks with no gene annotation. (**E**) Illustration of PM_2.5_ exposure–induced DNA accessibility peaks profiles of DARS in specific pathways of interest. Left: BAT Metabolism (*Ucp1*). Middle: ROS and Glutathione Metabolism (*Gclc*), Right: Circadian Rhythm (*Sik1*). (**F**) GO analysis (Biological Process) of DAR significant GO terms by DNA accessibility status. (**G** and **H**) Bar plots of GREAT analysis by DNA accessibility status. (**G**) Genomic partition distribution and (**H**) genomic feature distance distribution (DAR peaks to TSSs) of DARs associated with GA or LA. (**I** and **J**) Predicted DAR target motifs and regulatory enhancer sites by GREAT analysis. (**I**) Comparison of significant transcription factors motifs of DAR target genes by DNA accessibility status. (**J**) RGT_HINT analysis of significant (enriched) transcription factor DNA-binding sites of DAR target genes by DNA accessibility status.

Figure 4A shows a multidimensional scaling (MDS) plot. We identified a total of 2278 differentially accessible regions (DARs, see Methods), of which 833 were gain of accessibility (GA) (PM_2.5_ > FA) and 1445 were loss of accessibility (LA) (PM_2.5_ < FA) regions. After further reducing the dimensionality, a curated list of 1861 unique DARs was assembled (482 GA and 1379 LA, Supplemental Table 1B and Supplemental Figure 3). The plots in Figure 4, B and C show the distribution of significant DARs by DNA accessibility status and endorse the power of our variable selection method, as all DARs would have been called nonsignificant (false negative) had we used a regular fold-change method (e.g., |log_2_[FC]| > 2). Figure 4D identifies the top 10 GA and top 10 LA DARs, also mapped in the Bayesian ANOVA (BAM) “M” and volcano scatter plots (Figure 4, B and C). Figure 4E illustrates DNA accessibility profiles of representative genes of interest in the key pathways of BAT metabolism, ROS and glutathione synthesis, and circadian rhythm altered in response to PM_2.5_. A significant reduction in DNA accessibility in response to PM_2.5_ exposure, as evidenced by the average assay for transposase-accessible chromatin using sequencing (ATAC-seq) peak enrichment, was noted in *Ucp1*, *Gclc*, and *Sik1*. Figure 4F depicts the results of GO terms in Biological Process by DNA accessibility status. The DARs in the PM_2.5_ samples were primarily localized in intronic regions (Figure 4G) and were mostly in distal regulatory sites (5–500 kb) downstream of the TSS, with a much smaller proportion of upstream promoter sites that were differentially accessible (Figure 4H and Supplemental Figure 4). We also characterized the functional annotations of all identified DAR enhancer and regulatory sites predicted by Genomic Regions Enrichment of Annotations Tool (GREAT) analysis (see Methods) (24). In addition, we performed de novo motif prediction using HOMER software (see Supplemental Methods) to identify potential TFBSs that overlap with DARs. In the PM_2.5_ samples, DARs associated with GA were enriched in TFBSs that corresponded to *Stat5*, *Arid3a*, *Thrb*, and *Barx1*, while DARs associated with LA were enriched in TFBSs that corresponded to *Spf1*, *Klf15*, *Znf519*, *Hinfp*, and *Tfap2c* (Figure 4I). Through analysis using RGT_HINT software (25), we observed similarly enriched TFBSs, such as the *Spf*, *Klf*, and *Znf* families, in DARs primarily associated with LA (Figure 4J).

### Air pollution–induced mRNA transcription patterns in BAT.

**Figure 5:**
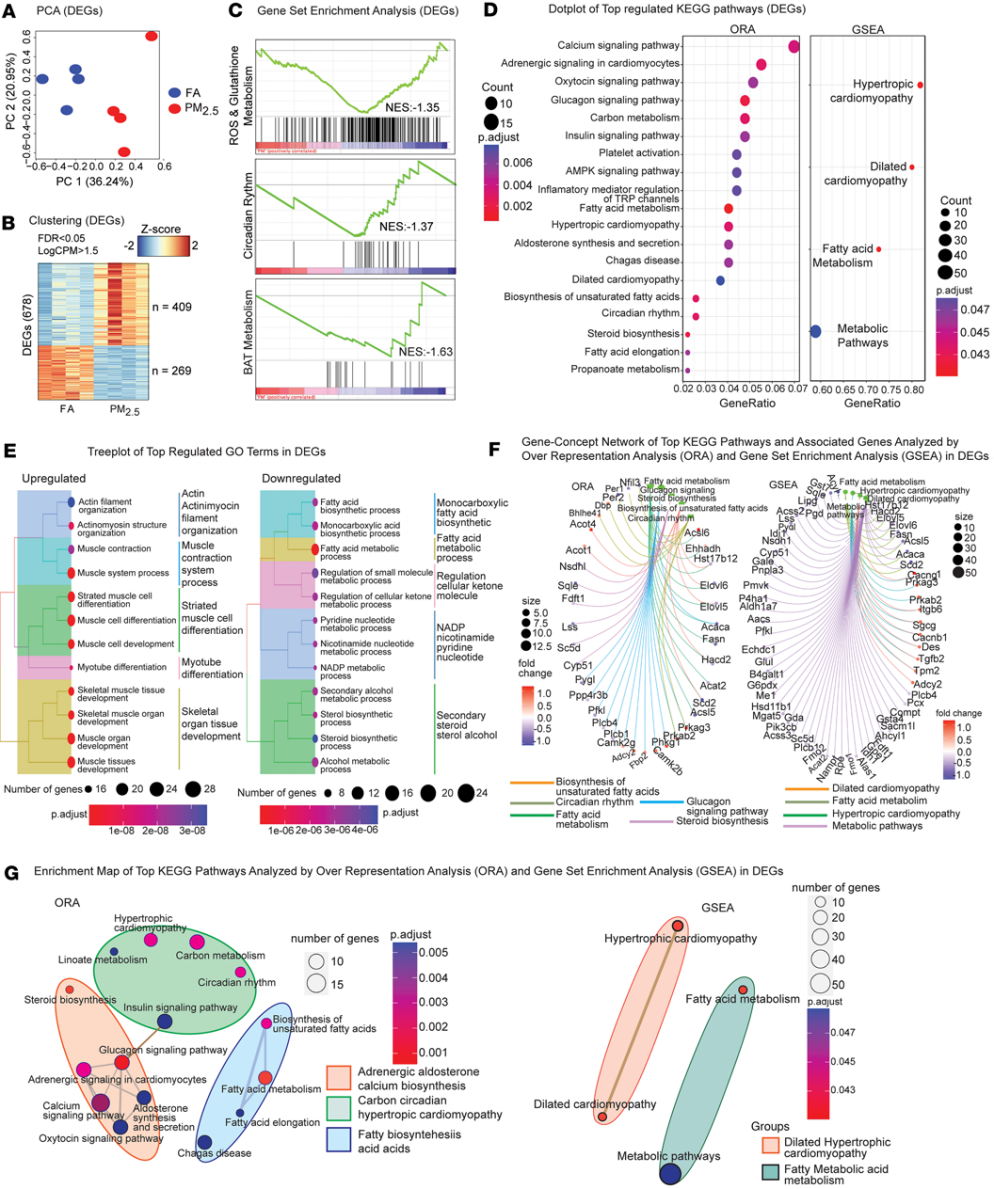
Transcriptome data analysis of PM_2.5_-exposed BAT: differential expression and associated functional analyses. (**A**) PCA plot of biological replicates from FA (*n* = 4) and PM_2.5_ (*n* = 4) in the transcriptome data of BAT. (**B**) Hierarchical clustering and heatmap plot of statistically significant differentially expressed genes (DEGs: 663, of which 409 are upregulated and 269 are downregulated) in PM_2.5_-exposed BAT. (**C**) GSEA of transcriptome data in specific pathways of interest. Left: ROS and Glutathione Metabolism. Middle: Circadian Rhythm. Right: BAT Metabolism. Red: upregulated; Blue: downregulated in PM_2.5_. NES, normalized enrichment score. Most of the genes in these pathways were downregulated. (**D**–**G**) GO and KEGG pathways of DEGs by overrepresentation analysis (ORA) and GSEA. (**D**) Dot plot of top regulated KEGG pathways of DEGs analyzed by ORA (left) and GSEA (right). (**E**) Tree plot views of significant (ORA enriched) top regulated GO terms. Left: Upregulated GO terms. Right: Downregulated GO terms. (**F**) Gene-concept network of top KEGG pathways (smallest pathways’ *P* values from **B**) and associated genes in DEGs. Left: ORA. Right: GSEA. (**G**) Enrichment map of top KEGG pathways in DEGs. Left: ORA. Right: GSEA. The thickness of an edge between any 2 KEGG pathways is proportional to the overlap between the 2 pathways.

Figure 5, A and B show the resulting PCA plot and heatmap after clustering, respectively. We identified a total of 678 differentially expressed genes (DEGs). Out of these DEGs, 409 were found to be upregulated (PM_2.5_ > FA) while 269 were downregulated (PM_2.5_ < FA). After further reduction of dimensionality of the data, by selecting out the features with redundant or missing gene annotations, a curated list of 663 fully annotated DEGs was obtained (402 upregulated and 261 downregulated, Supplemental Table 1C). The top upregulated genes included *Rreb1*, *Ucp3*, *Tsku*, *Ky*, and *Vamp1*, while top downregulated genes included *Klf15*, *Gm45061*, *Fam13a*, *Zcchc2*, *Ccng2*, *Ppp1r3b*, *Slc5a3*, and *Gas1*. Gene set enrichment analysis (GSEA) identified ROS, glutathione, circadian rhythm, and BAT metabolism pathways to be downregulated in the PM_2.5_ (Figure 5C). Panels D–G of Figure 5 give a more in-depth view of the DEG functions and activated pathways. Figure 5D shows the results for the Kyoto Encyclopedia of Genes and Genomes (KEGG) pathways of DEGs generated through overrepresentation analysis (ORA) and GSEA. Among the pathways regulated in the PM_2.5_-exposed mice, calcium signaling, insulin signaling, fatty acid metabolism, and circadian rhythm were prominent. The PM_2.5_ samples exhibited a significant upregulation of genes associated with muscle development and actin cytoskeleton organization, and significant downregulation in BAT lipid biosynthetic and fatty acid metabolic processes. The tree view of Figure 5E shows the hierarchy of enriched GO terms from up- and downregulated DEGs. The gene-concept network view reveals which important genes are involved in the top KEGG pathways (by significance of *P* values, Figure 5F), while the enrichment map highlights the relationships and overlapping areas that exist between any 2 top KEGG pathways (Figure 5G).

### DMR and DEG integration analyses by MPLS.

**Figure 6:**
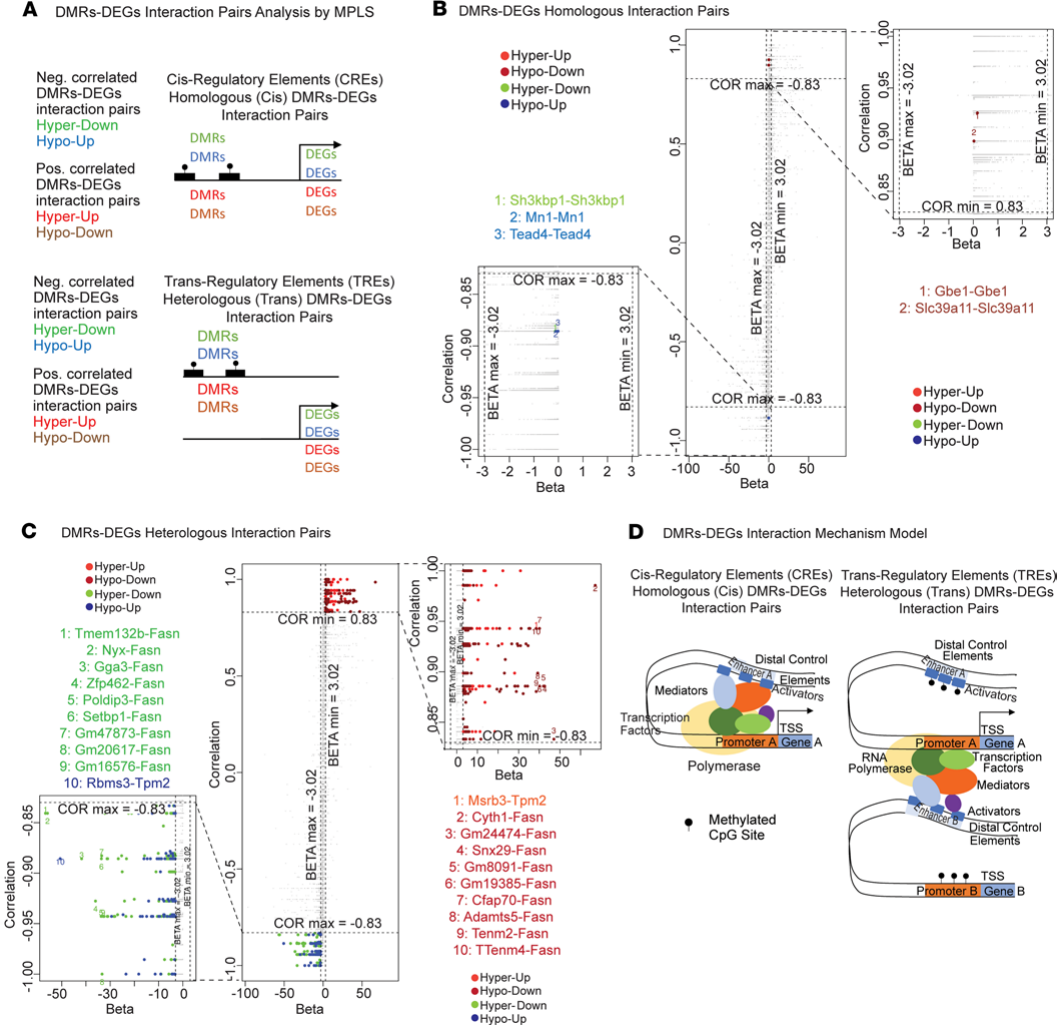
DNA accessibility data analysis of PM_2.5_-exposed BAT and integrative analysis with transcriptome data: overall integrative analysis. Integrative analysis of transcriptome and methylome data on curated DMR-DEG interaction pairs identified by MPLS (see Methods). (**A**) Categories of DMR-DEG interaction pairs by interaction type (homologous [top] vs. heterologous [bottom]), directionality of change (between hypermethylated and hypomethylated DMRs and up- vs. downregulated DEGs) and correlation sign (between regression and correlation coefficients). (**B** and **C**) Scatter plots of DMR-DEG interaction pairs in the correlation-regression space (**B**, homologous vs. **C**, heterologous). For each interaction type, a point represents a DMR-DEG pair. Correlation and regression coefficient thresholds of significance are shown (in-plot dotted lines). Full (middle plot) and close-up views (left- and right-hand sides of plots) of all significant pairs are highlighted and mapped on the plots. Lists of up to top 10 significant pairs are shown. (**B**) Top 5 homologous DMR-DEG pairs. Left: 3 homologous negatively correlated DMR-DEG pairs. Right: 2 homologous positively correlated DMR-DEG pairs. (**C**). Top 616 heterologous DMR-DEG pairs. Left: 319 heterologous negatively correlated DMR-DEG pairs. Right: 297 heterologous positively correlated DMR-DEG pairs. (**D**) DMR-DEG interaction mechanism model. Left: *Cis* regulatory elements (CREs) corresponding to homologous (*cis*) DMR-DEG pairs. Right: *Trans* regulatory elements (TREs) corresponding to heterologous (*trans*) DMR-DEG pairs.

We used a joint approach of correlation analysis and multivariate partial least squares (MPLS) modeling (see Supplemental Methods) (26). We identified 621 DMR-DEG significant interaction pairs. Five of these pairs were homologous (*cis*), meaning that the methylation occurred in the promoter or enhancer of the same DEG (Figure 6, A and B, and Supplemental Table 2A), while 616 were heterologous (*trans*), meaning that the methylations occurring in promoter/enhancers of these genes were strongly correlated with differential expression of other genes (Figure 6, A and C, and Supplemental Table 2B). When all DMR-DEG pairs are plotted in the correlation coefficient versus regression coefficient space for visualization, one notices that inferences from straight correlation analysis and MPLS modeling are consistent; almost all DMR-DEG pairs are found in the upper-right and lower-left quadrants of the correlation-regression space (Figure 6B). For reasons of inherent mathematical imbalance between the number of homologous and heterologous pairs, the homologous pairs were called significant by one criterion of correlation or regression, while the heterologous pairs were called significant by both criteria (see Supplemental Methods). As a result, significant homologous pairs appear beyond one threshold of significance (top or bottom of the correlation-regression space, Figure 6B), while significant heterologous pairs appear beyond both thresholds of significance (upper-right or bottom-left corners of the correlation-regression space, Figure 6C). Intriguingly, a very large majority of significant DMR-DEG interaction pairs involved heterologous interaction pairs (Figure 6, A and C, and Supplemental Table 2, A and B). Figure 6D represents a hypothetical model of DMR-DEG interactions.

KEGG pathway analysis of homologous and heterologous DMR-DEG pairs revealed enrichment in pathways such as Striated Muscle Functions, Development, and Adaptation, especially in relation to cardiac muscle and cardiac muscle hypertrophy in response to stress. Of note was the activation of pathways such as Insulin Signaling/Insulin Resistance and Fatty Acid Metabolism Biosynthesis (Supplemental Table 3, A and B, highlighted in red).

Next, we searched for significant (enriched) blocks or regions of differentially methylated CpG sites within promoters and enhancers (±2 kb of the TSS) of corresponding gene expression changes, i.e., significant DEGs. Results are shown in a scatter plot and contingency table of all significant interaction pairs (Supplemental Figure 5A). Overall, a strong dependence was observed between the 2 assays; an anticorrelation trend was observed between gene methylation changes and gene expression changes (Supplemental Figure 5A, DNA methylation log[FC] vs. mRNA log[FC] χ^2^ ≈ 999.6, *P* ≈ 2.1 × 10^–219^).

### DAR and DEG integration analyses by MPLS modeling.

**Figure 7:**
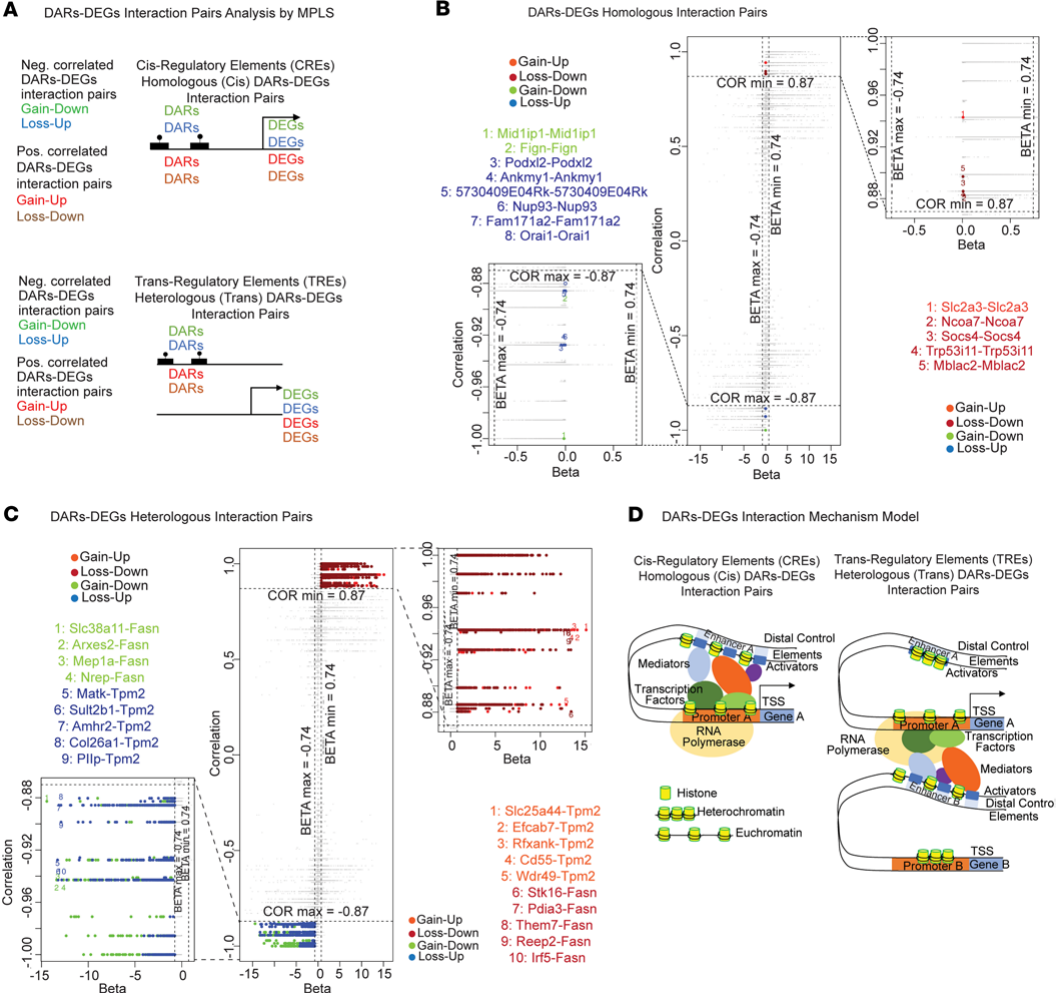
DNA accessibility data analysis of PM_2.5_-exposed BAT and integrative analysis with transcriptome data: overall integrative analysis. Integrative analysis of transcriptome and DNA accessibility data on curated DAR-DEG interaction pairs identified by MPLS (see Methods). (**A**) Categories of DAR-DEG interaction pairs by interaction type (homologous [top] vs. heterologous bottom]), directionality of change (between gain of accessibility [GA] and loss of accessibility [LA] DARs and up- vs. downregulated DEGs) and correlation sign (between regression and correlation coefficients). (**B** and **C**) Scatter plots of DAR-DEG interaction pairs in the correlation-regression space (**B**, homologous vs. **C**, heterologous). For each interaction type, a point represents a DAR-DEG pair. Correlation and regression coefficient thresholds of significance are shown (in-plot dotted lines). Full (middle plot) and close-up views (left and right-hand sides plots) of all significant pairs are highlighted and mapped on the plots. Lists of up to top 10 significant pairs are shown. (**B**) Top 13 homologous DAR-DEG pairs. Left: 8 homologous negatively correlated DAR-DEG pairs. Right: 5 homologous positively correlated DAR-DEG pairs. (**C**). Top 4242 heterologous DAR-DEG pairs. Left: 1996 heterologous negatively correlated DAR-DEG pairs; Right: 2246 heterologous positively correlated DMR-DEG pairs. (**D**) DAR-DEG interaction mechanism model: Left: *Cis* regulatory elements (CREs) corresponding to homologous (*cis*) DAR-DEG pairs. Right: *Trans* regulatory elements (TREs) corresponding to heterologous (*trans*) DAR-DEG pairs.

To study how genomic regions of DNA accessibility (blocks of peaks) regulate downstream gene expression, we used the curated DAR (Supplemental Table 1B) and DEG datasets (Supplemental Table 1C, see also Supplemental Methods). Using the same joint approach of correlation analysis and MPLS regression modeling for data (see Supplemental Methods) (26), we identified 4255 DAR-DEG significant interaction pairs (13 homologous/*cis*, Figure 7, A and B, and Supplemental Table 4A; 4242 heterologous/*trans*, Figure 7, A and C, and Supplemental Table 4B). The conclusions were similar to those for the MPLS modeling of DMR-DEG interactions, including the fact that the very large majority of significant DAR-DEG interactions were heterologous (Figure 7, A and C, and Supplemental Table 4, A and B). GO and KEGG pathway databases revealed enrichment in several pathways such as T2D, Insulin Signaling and Secretion, and Fatty Acid Biosynthesis and Metabolism. Of note is also the activation of *JAK-STAT*, *MAPK*, *AMPK*, and *cAMP* signaling pathways, among others (Supplemental Table 5, A and B, highlighted in red). Figure 7D represents a hypothetical model of DAR-DEG interactions.

We also searched for significant (enriched) blocks or regions of differentially accessible chromatin peaks within promoters and enhancers (±2 kb of the TSS) of corresponding gene expression changes, i.e., significant DEGs. Results are shown in a scatter plot and contingency table of all significant interaction pairs (Supplemental Figure 5B) and comparison was done (Supplemental Figure 5B) by comparing the counts by categories using a χ^2^ test of independence (Supplemental Figure 5B). Overall, a strong dependency was observed between the 2 assays; a correlation trend was observed between gene accessibility changes and gene expression changes (Supplemental Figure 5B, DNA accessibility log[FC] vs. mRNA log[FC] χ^2^ ≈ 2090.7, *P* < 10^–300^).

### Overall DMR, DAR, and DEG integration analyses.

**Figure 8:**
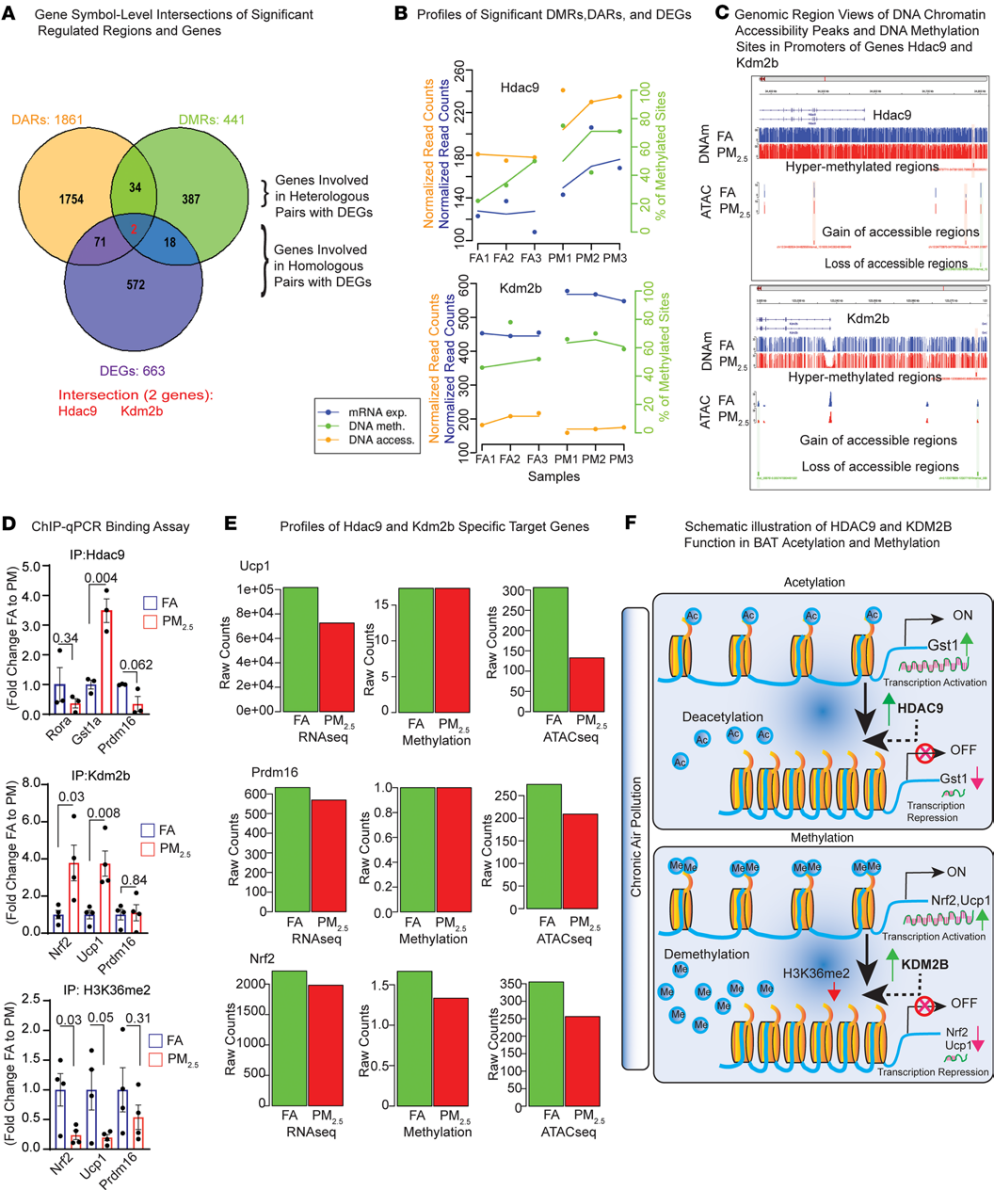
DNA accessibility data analysis of PM_2.5_-exposed BAT and integrative analysis with transcriptome data: overall integrative analysis. (**A**) Venn diagram of gene-level intersection analyses. Intersections of significant genes and regions as well as target genes predicted by GREAT, ATLAS, and MPLS analyses on curated interaction pairs (see Methods). Three-set intersections between all significant genes and regions (DEGs, 663; DMRs, 441; DARs, 1861). Listed in red are the 2 genes (*Hdac9* and *Kdm2b*) found significant in all 3 assays (DMRs, DARs, and DEGs). (**B**) Profiles of DNA chromatin accessibility levels (orange, normalized read counts within region), DNA methylation levels (green, percentage of methylated sites over total sites [mCpG/CpG] within region), and mRNA expression levels (blue, normalized read counts within region) of the 2 genes (*Hdac9* and *Kdm2b*) found to be significant in all 3 assays (DMRs, DARs, and DEGs). Top: *Hdac9*. Bottom: *Kdm2b*. (**C**) Genomic region views of DNA chromatin accessibility peaks and DNA methylation sites in the promoters of genes *Hdac9* and *Kdm2b*. Blue, FA group; Red, PM_2.5_ group. *Hdac9* top: right-to-left ORF (cropped, negative DNA strand). *Hdac9* middle: DNA methylated sites showing one significant hypermethylated DMR (red rectangle). *Hdac9* bottom: DNA accessibility peaks showing one significant gain of accessibility (GA) and 2 loss of accessibility (LA) DARs (red and green rectangles). *Kdm2b* top: right-to-left ORF (negative DNA strand). *Kdm2b* middle: DNA methylated sites showing one significant hypermethylated DMR (red rectangle). *Kdm2b* bottom: DNA accessible peaks showing 2 significant LA DARs (green rectangles). (**D**) ChIP-qPCR binding assay. Top: *Hdac9* (*n* = 3) binding sites on *Rora*, *Gst1a*, and *Prdm16* promoters. Middle: *Kdm2b* (*n* = 4) binding sites on *Nrf2*, *Ucp1*, and *Prdm16* promoters. Bottom: *H3k36me2* (*n* = 4) binding sites on *Nrf2*, *Ucp1*, and *Prdm16* promoters. The fold changes of binding regions between exposure groups are indicated. See also Supplemental Table 9. (**E**) Whole genome profiles of *Hdac9* and *Kdm2b* specific target genes: *Ucp1* (top), *Prdm16* (middle), and *Nrf2* (bottom). (**F**) Schematic diagram illustrating the PM-induced *Hdac9*-associated deacetylation and corresponding gene downregulation, and PM-induced *Kdm2b*-associated demethylation mechanism and corresponding gene downregulation. Data are provided as mean ± SEM. *P* values versus FA-exposed mice by unpaired, 2-tailed Student’s *t* test.

The significantly regulated regions (441 DMRs, Supplemental Table 1A; and 1861 DARs, Supplemental Table 1B) and genes (663 DEGs, Supplemental Table 1C) identified from the curated datasets, as well as significant DMR-DEG and DAR-DEG interaction pairs identified either by MPLS, ATLAS (Supplemental Methods), or GREAT analysis were used to conduct gene-level intersection analyses. We first show intersections (or not) of significant regions of DARs, DMRs, and DEGs in Figure 8A. This resulted in 73 homologous and 1788 heterologous regions and genes involved in DAR-DEG interaction pairs, as well as 20 homologous and 421 heterologous regions and genes involved in DMR-DEG interaction pairs. Out of these, only 2 (*Hdac9* and *Kdm2b*) were common to both DAR-DEG and DMR-DEG interaction pairs, meaning that they are simultaneously regulated at the DNA accessibility, methylation, and mRNA expression levels (Figure 8A). The lists of unique and significant DAR-DEG and DMR-DEG interaction pairs found by MPLS integration analyses (Figure 6, A and C, Figure 7, A and C, Supplemental Table 2, A and B, and Supplemental Table 4, A and B) were subjected to similar gene-level intersection analyses. Supplemental Figure 6A verified that no (zero) homologous pair intersected with a heterologous pair and revealed that very few (only 9) of the DAR-DEG and DMR-DEG interaction pairs intersect with each other.

Supplemental Figure 6 shows corresponding intersections of significant pairs. To confirm, we also matched the target genes predicted by GREAT or ATLAS analyses with MPLS modeling. Gene-level intersections of significant interaction pairs predicted by MPLS and GREAT are shown in Supplemental Figure 6B. Focusing on the 5 central intersections (highlighted in red, Supplemental Figure 6B), 14 target genes common to DARs and DMRs were confirmed by at least 2 independent analytical methods (GREAT and MPLS) in at least one assay (DNA chromatin accessibility or methylation). These 14 genes (listed in red, Supplemental Figure 6B), are reported in Supplemental Table 6, where each row is a DMR-DEG (top) or DAR-DEG (bottom) interaction pair containing one instance of these genes and showing the expression level of the corresponding DEG. Similarly, gene-level intersections of significant interaction pairs predicted by MPLS and ATLAS are shown in Supplemental Figure 6C. Here, focusing on the 5 central intersections (highlighted in red, Supplemental Figure 6C), 13 target genes common to DARs and DMRs were confirmed by at least 2 independent analytical methods (ATLAS and MPLS) in one assay (DNA chromatin accessibility or methylation). Similarly, these 13 genes (listed in red, Supplemental Figure 6C) are reported in Supplemental Table 7, where each row is a DMR-DEG (top) or DAR-DEG (bottom) interaction pair containing at least one instance of these genes and showing the expression level of the corresponding DEG.

Furthermore, we used ATLAS analysis for the intersection of predicted enhancer sites from 2 epigenome data analytical studies (Supplemental Figure 7A) to determine the genomic distance from DMR sites or DAR peaks to target enhancers. We show histograms of distributions of genomic distances by assay with the overlap between ATLAS and MPLS predictions by bins (Supplemental Figure 7B). For each overlap, the hypergeometric test was applied, and all overlaps were found to be highly significant (Supplemental Figure 7B; hypergeometric test *P* values from proximal to distal bins, DMRs: *P* ≈ 7.7 × 10^–17^, *P* ≈ 2.5 × 10^–33^, *P* ≈ 1.8 × 10^–16^, *P* ≈ 5.1 × 10^–13^; DARs: *P* ≈ 4.1 × 10^–47^, *P* ≈ 1.3 × 10^–64^, *P* ≈ 1.2 × 10^–33^, *P* ≈ 7.9 × 10^–36^).

The above DMRs and DARs common target genes (14 and 13) were pooled together and included in an in-depth functional analysis (Supplemental Figure 8). Supplemental Figure 8A shows the result of enriched GO terms of DMR and DAR common target genes analyzed by ORA. Among the significant ontologies activated in PM_2.5_-exposed mice, one notes the significance of RNA Carbohydrate Domain DNA-Binding, *Atg8* and *Atg12* Activating Enzymes, and Epidermal *Erb-3* and *Erb-4* Class Receptor Binding ontologies in PM_2.5_-exposed mice. The tree view gives the corresponding hierarchy of enriched GO terms (Supplemental Figure 8B), the gene-concept network view reveals which important genes are involved with (sometimes multiple) top GO terms (Supplemental Figure 8C), while the enrichment map highlights relationships and overlaps that exist between any 2 GO terms (Supplemental Figure 8D).

Based on our DMR, DAR, and DEG interaction analysis, we identified *Hdac9* and *Kdm2b* as genes that intersect in all categories (Figure 8A, Supplemental Figure 9, and Supplemental Table 9). Figure 8B shows their corresponding patterns of mRNA expression, DNA chromatin accessibility, and DNA methylation and Figure 8C shows the corresponding patterns of DNA chromatin accessibility and DNA methylation sites in their promoters. We performed a binding assay of HDAC9, KDM2B, and H3K36me2 with limited target genes of interest, and compared results between BAT of PM_2.5_ and FA mice. Immunoprecipitated HDAC9 physically interacted with *Prdm16*, *Rora*, and *Gst1a* (Figure 8D) but only *Gst1a* binding was increased (Figure 8D). It is conceivable that HDAC9 interaction promotes decreased expression of *Gst1a* in BAT of PM_2.5_ mice; transcriptional repression of *Gst1a* by HDAC9 binding may drive oxidative stress in BAT. HDAC9 also physically interacted with *Prdm16* and *Rora*, resulting in their downregulation, although this was not significant (Figure 8D). We detected a significant 2-fold increased binding of *Kdm2b* to *Nrf2* and *Ucp1* upon PM_2.5_ exposure (Figure 8D). KDM2B is a histone lysine demethylase, which targets dimethyl residues for demethylation. *H3K36me2* methylation status can be used as a marker for KDM2B activity. Reduced *H3K36me2* binding was detected in *Nrf2* and *Ucp1* in PM_2.5_ mice (Figure 8D), suggesting that KDM2B represses *Nrf2* and *Ucp1* transcription through increased di-demethylation in histone residues across the genome. The reduced methylation at the *H3K36me2* histone mark is surrogate evidence of potential KDM2B activity in PM_2.5_ mice (Figure 8D). To further confirm our findings, we analyzed our RNA-seq, whole-genome bisulfite sequencing (WGBS), and ATAC-seq profiles to assess potential changes in the target genes of HDAC9 and KDM2B: *Ucp1*, *Prdm16*, and *Nrf2*. We observed decreased raw counts of *Ucp1* in RNA-seq, which correlated with reduced raw counts in ATAC-seq, although methylation levels remained unchanged (Figure 8E). Similarly, the raw counts for *Prdm16* in both RNA-seq and ATAC-seq were consistent, but methylation did not show any changes. Interestingly, the raw counts for RNA seq, methylation, and ATAC-seq of *Nrf2* were all decreased, indicating reduced chromatin accessibility, which corresponds to the downregulation of transcription in *Ucp1*, *Prdm16*, and *Nrf2* (Figure 8E). Figure 8F illustrates the mechanism of HDAC9 and KDM2B function in BAT acetylation and methylation, respectively.

**Figure 9:**
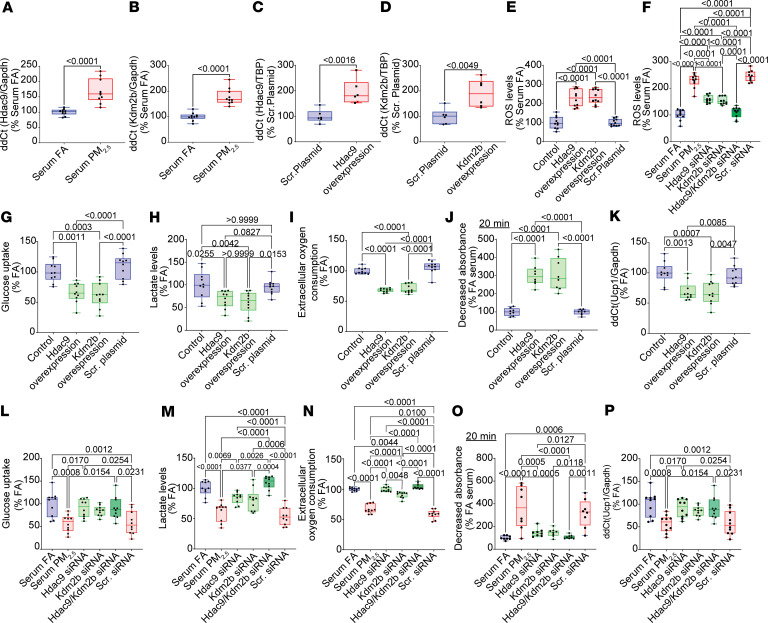
Effect of siRNA-mediated knockdown and CRISPR/Cas9-mediated overexpression of *Hdac9* and *Kdm2b* on ROS generation, bioenergetics, and mitochondrial function in BAT cells in vitro. (**A**) *Hdac9* and (**B**) *Kdm2b* mRNA levels were analyzed by qRT-PCR in BAT cells treated with serum derived from FA- and PM_2.5_-exposed mice for 48 hours. (**C**) *Hdac9* and (**D**) *Kdm2b* mRNA levels were analyzed by qRT-PCR in BAT cells overexpressing either a scrambled plasmid or *Hdac9* or *Kdm2b*. TBP was used as an internal control. (**E**) ROS production levels in BAT cells overexpressing *Hdac9*, *Kdm2b*, or a scrambled plasmid (control). (**F**) ROS production levels in BAT cells transfected with either control (scrRNA) or *Hdac9* or *Kdm2b* siRNA and treated with serum from FA- and PM_2.5_-exposed mice for 48 hours. (**G**) Glucose uptake, (**H**) lactate, (**I**) ECR, (**J**) mitochondrial swelling, and (**K**) *Ucp1* mRNA level in BAT cells overexpressing *Hdac9*, *Kdm2b*, or a scrambled plasmid (control). (**L**) Glucose uptake, (**M**) lactate, (**N**) ECR, (**O**) mitochondrial swelling, and (**P**) *Ucp1* mRNA level in BAT cells transfected with either control (scrRNA) or *Hdac9* or *Kdm2b* siRNA and treated with serum from FA- and PM_2.5_-exposed mice for 48 hours. *P* values were calculated by unpaired Student’s *t* test for 2 group comparisons and 1-way ANOVAs (Bonferroni’s multiple comparison) for comparisons involving 3 or more groups. The data were obtained from 3 independent experiments.

Since we have demonstrated that *Hdac9* and *Kdm2b* are highly expressed in BAT upon exposure to PM_2.5_, and that their expression is positively correlated with a decrease in *Nrf2* expression (an upstream regulator of antioxidant activity) and *Ucp1* (a crucial regulator of BAT thermogenesis), we aimed to confirm the roles of *Hdac9* and *Kdm2b* in BAT oxidative stress and bioenergetics in brown adipocytes (T37i cell line). To achieve this, we performed transient overexpression and knockdown of these epigenetic regulators. BAT cells treated with serum from PM_2.5_-exposed mice exhibited a significant upregulation of *Hdac9* and *Kdm2b* expression (Figure 9, A and B) compared with serum from FA-treated cells. Also, transient overexpression of *Hdac9* and *Kdm2b* effectively enhanced their expression at the mRNA level in BAT cells (Figure 9, C and D) relative to cells transfected with a scrambled plasmid. Moreover, overexpression of these genes led to a marked increase in oxidative stress (Figure 9E), which was subsequently reduced by treatment with small interfering RNAs (siRNAs) targeting the mRNA regions of *Hdac9* and *Kdm2b* (Figure 9F). In our bioenergetic studies, transient overexpression of either *Hdac9* or *Kdm2b* (Figure 9, G–I) resulted in a significant decrease in glucose uptake, lactate production, and extracellular oxygen consumption (ECR), while simultaneously promoting mitochondrial swelling (Figure 9J and Supplemental Figure 10). These changes were associated with a reduction in *Ucp1* expression (Figure 9K). In contrast, knockdown of either *Hdac9* or *Kdm2b* led to a significant increase in glucose uptake, lactate levels, ECR, and a reduction in mitochondrial swelling (Figure 9, L–O, and Supplemental Figure 10) compared with scrambled RNA–treated cells. These effects corresponded to an increase in *Ucp1* expression (Figure 9P). The above effects were more pronounced when both genes were silenced simultaneously in BAT cells. Taken together, these results suggest that PM_2.5_ exposure suppresses key processes such as antioxidant activity and mitochondrial bioenergetics in BAT via distinct epigenetic pathways mediated through *Hdac9* and *Kdm2b*.

## Discussion

We have demonstrated an important effect of chronic ambient PM_2.5_ exposure on BAT function and whole-body metabolism resulting from redox stress, lipid deposition, and reduced thermogenic pathways. These effects were associated with widespread chromatic remodeling and genome-wide differential methylation, involving sites enriched for TFBSs, including circadian and *Nrf2* targets. Changes in chromatin accessibility and differential methylation mostly involved enhancer elements in distinct genes (heterologous). Only 2 genes, *Hdac9* and *Kdm2b*, were regulated through simultaneous changes in mRNA expression, chromatin accessibility, and DNA methylation with ChIP-qPCR for *H3K36me2*, revealing that HDAC9 physically interacted with multiple genes involved in redox stress, circadian rhythm, and BAT metabolism, with reduced expression of *Nrf2*, *Ucp1*, and *Prdm16*. We demonstrate that plasma from PM_2.5_-exposed mice increased expression of *Hdac9* and *Kdm2b* in cultured brown adipocytes, with overexpression of *Hdac9* and *Kdm2b* lowering *Ucp1* and reducing glucose uptake, lactate production, and oxygen consumption, while silencing both genes improved bioenergetics and increased *Ucp1*. These effects were exaggerated when both *Hdac9* and *Kdm2b* genes were silenced together.

Impaired glucose clearance and hyperglycemia and reduced insulin-induced glucose uptake in BAT, detected by FDG distribution, were associated with reduced RER and whole-body energy expenditure with PM_2.5_. BAT tissue from PM_2.5_-exposed mice exhibited increases in redox stress, lipid deposition, fibrosis, changes in circadian rhythmicity and BAT metabolic genes, and a reduction in antiinflammatory cytokines. BAT ultrastructural changes included reductions in mitochondrial number and alterations in mitochondrial cristal architecture were noted, which have been previously described in response to PM_2.5_ exposure (20, 21). Collagen deposition in BAT sections was enhanced together with an increase in adipocyte mRNA levels of *Angptl2*, a gene enhancing fibrosis accumulation by elevating *Tgfb*, while *Gtf2ird1*, a gene responsible for repressing adipose tissue fibrosis, was reduced (27–29).

Widespread changes in chromatin accessibility in response to air pollution exposure was noted, with a refined set of 1861 DARs (482 GA, 1379 LA). Most DARs were in intronic and distal regulatory regions rather than promoter regions. GO analysis of DARs revealed enrichment in pathways related to ER stress, tyrosine dephosphorylation, and stress-activated protein kinase signaling, accompanied by a gain in chromatin accessibility. These pathways are integral to the hallmark features of metabolic dysregulation observed in PM_2.5_-exposed mice. Key genes involved in BAT metabolism (*Ucp1*), oxidative stress response (*Gclc*), and circadian regulation (*Sik1*) showed reduced accessibility following PM_2.5_ exposure. Motif analysis revealed that GA DARs were enriched for TFBSs, such as STAT5 and ARID3a, while LA DARs were enriched for TFBSs including SPF1, KLF15, and ZNF519. Increased enrichment of *Stat5*-binding sites in GA DARs suggests enhanced activation of inflammatory signaling pathways — likely a stress response to PM_2.5_ that could dampen BAT’s thermogenic function, while enrichment of *Arid3a* motifs may reflect epigenetic remodeling — potentially suppressing genes involved in mitochondrial biogenesis or thermogenic activation, both essential to BAT function. In contrast, loss of accessibility to *Klf15* is a key metabolic regulator, promoting lipid oxidation and thermogenic gene expression in BAT. It also has antiinflammatory effects. Loss of accessibility at *Klf15* sites likely reflects a shutdown of BAT’s thermogenic and antiinflammatory programs, shifting the tissue toward metabolic inefficiency and inflammation.

To investigate how changes in chromatin accessibility affect gene expression, we integrated DARs with DEGs using correlation and MPLS regression. This analysis revealed 4255 significant DAR-DEG pairs, with the vast majority occurring in *trans* rather than *cis* pairs. Enriched pathways included insulin signaling, fatty acid metabolism, and key regulatory cascades like JAK-STAT, MAPK, AMPK, and cAMP. DARs within promoter/enhancer regions (±2 kb of TSS) of genes correlated with gene expression changes with a strong dependency between accessibility and expression changes (χ^2^ ≈ 2090.7, *P* < 10^–300^), supporting a coordinated regulatory relationship.

Exposure to PM_2.5_ significantly altered DNA methylation patterns in BAT, with 881 DMRs identified —roughly split between hypermethylated and hypomethylated sites. Most DMRs were found in genomic regions distant from gene promoters, such as introns, intergenic areas, and repetitive elements (LINEs and LTRs). An important finding in our study was the overrepresentation of TFBSs of genes in response to PM_2.5_. This included T cell factor 7 (*Tcf7*), NF-κB p65 (*Nfkbp65*), BARX homeobox 1 (*Barx1*), ETS transcription factor ELK4 (*Elk4*), and NK2 homeobox 2 (*Nkx2*). These TFBSs were enriched in hypermethylated DMRs, while the TFBSs of *Foxo1*, *Elf3*, *Foxh1*, *Cdx1*, *Smad1*, and *Prdm15* were enriched in hypomethylated DMRs. Hypomethylated regions were associated with genes involved in enhanced gluconeogenesis and inflammation, while hypermethylated regions mapped to pathways linked to metabolic dysregulation, insulin resistance (*Lep*, *Adipoq*, *Mapk10*) and antioxidant dysfunction via reduced levels of NRF2 (*Serpine1*, *Id2*) (30). TCF7 binds to specific DNA sequences and acts as a transcription factor, and plays a critical role in regulating gene expression (31). PRDM16, a key regulator of brown adipocyte differentiation, collaborates with TCF7 to mediate the transcription of *Ucp1*, a gene essential for nonshivering thermogenesis (32). An upregulated NF-κB pathway heightens inflammation but also can enhance oxidative stress by promoting *Keap1* expression (which degrades *Nrf2*) (33).

In our integration analysis of DEGs, DARs and DMRs, we uncovered only 2 targets, *Hdac9* and the histone demethylase *Kdm2b* that were upregulated. Their roles in the regulation of other BAT transcription factors, antioxidant transcription factors, and circadian targets were further confirmed using ChIP-qPCR and *H3K36me2* methylation status. Our results are supportive of a physical interaction between *Hdac9* and *Prdm16*/*Rorα*/*Gst1* and suggest a close cooperation between epigenetic regulators and transcription factors involved in BAT thermogenesis and circadian rhythm. A previous report showed that *Hdac9* knockdown and overexpression influenced the expression of *Ucp1*, *Prdm16*, and *Nrf2*, supportive of our findings (34–36). *Prdm16* has been previously shown to physically interact with *Hdac* after treatment with *Hdac3*-selective inhibitors, inducing thermogenic gene expression (34). We found a trend toward reduced binding of *Hdac9* with *Prdm16* after PM_2.5_ exposure, which might be explained by *Hdac9* possessing differential effects, whereby it may either repress or activate *Prdm16* (37). We observed an increased expression of *Kdm2b* in PM_2.5_ mice that specifically demethylates *H3k36me2*, leading to repressive functions. Methylation at H3K4, H3K36, and H3K79 is usually associated with gene activation, whereas methylation at H3K9, H3K27, and H4K20 is associated with gene silencing (37). Our ChIP-qPCR results indicate *Kdm2b* strongly bound with the promoter of *Nrf2* and *Ucp1* in the BAT of PM_2.5_-exposed mice, suggesting *Kdm2b*-induced demethylation may decrease the expression of *Nrf2* and *Ucp1* in BAT (38, 39). *H3K36me2* significantly decreased in the promoters of *Nrf2* and *Ucp1* in PM_2.5_-exposed mice, implicating *Kdm2b* in the depletion of *H3K36* methylation, in turn leading to the transcriptional repression of these genes. Other studies have demonstrated that activation of *Kdm2b* reduced BAT-selective genes *Ucp1* and *Pgc1a* (39), while depletion of histone H3K36 methylation by *H3K36me2* impairs the induction of *Pparg* target genes, including *Ucp1* during adipogenesis (38). In our study, the *H3K36me2* status of *Prdm16* after PM_2.5_ exposure was comparable to FA, indicating *H3K36me2* depletion may not alter *Prdm16*. A previous study found a demethylase-independent role for *Kdm2b* in transcriptional repression through shaping the RNA polymerase II occupancy region (40). Thus, *Kdm2b* may also play a demethylase-independent role in *Prdm16* expression.

The deacetylation and demethylation of histones may serve as a tandem molecular switch to regulate brown adipose gene expression and differentiation. Under physiological circumstances, *Kdm2b* and *Hdac9* may be downregulated, with a facilitatory impact on the expression of multiple BAT targets. *Hdac9* and *Kdm2b* are upregulated in response to PM_2.5_, resulting in reduced DNA accessibility with repression of targets such as *Nrf2*, *Ucp1*, and *Prdm16* and adverse impacts on BAT metabolism. Deacetylation may further regulate methylation, as the absence of acetyl groups on lysines means that they can be methylated and recruit methylysine-binding proteins, some of which may counteract transcription (41). Indeed, deacetylated histones contribute to chromatin compaction through strengthening histone tail–DNA interactions (42, 43).

Exposure of cultured brown adipocytes to plasma from PM_2.5_-exposed animals recapitulated the effects of long-term PM_2.5_ exposure on BAT, with increased *Hdac9* and *Kdm2b* expression together with a reduction in *Ucp1* and BAT metabolic pathways. siRNA directed against either *Hdac9* or *Kdm2b* restored the impairment in BAT respiratory function, with silencing of both genes further enhancing effects. Based on our findings, we propose that chronic air pollution exposure modulates critical regulators such as *Hdac9* and *Kdm2b*, which in turn downregulate key genes like *Nrf2* and *Ucp1*, altering BAT metabolism and function. This cascade of events in response to PM_2.5_ enhances oxidative stress and shifts mitochondrial energy imbalance, leading to mitochondrial dysfunction in BAT, inflammation, and reduced metabolic function. Taken together, our findings provide insights into how air pollution contributes to metabolic dysfunction and may set the stage for development of T2D. Importantly, our study provides evidence for substantial redundancy in DNA methylation and chromatin accessibility, with most changes being transcriptionally silent. Our study has nonetheless some limitations that are worth acknowledging. The epigenetic changes in this study were at a single time point and to what extent these represent changes at other time points may need further investigation. Finally, further research is necessary to determine the extent to which these findings translate into metabolic changes in humans and in populations exposed to high levels of air pollution.

In conclusion, chronically inhaled PM_2.5_ impacts BAT function and metabolism through differential DNA methylation and impact on global chromatin structure. We demonstrate a potential causal role of 2 key epigenetic factors, *Hdac9* and *Kdm2b*, which may represent unique pathways through which air pollution exposure could alter BAT function.

## Methods

Further information can be found in Supplemental Methods.

### Sex as a biological variable.

In this study, we exclusively used male mice because they display less variability in phenotype; it is unknown whether the findings are relevant for female mice. Additionally, our previous study demonstrated a sex-dimorphic effect when exposed to air pollution (3). Consequently, female mice were excluded from the current study.

### Experimental design and air pollution exposure.

Male C57BL/6J mice (3 weeks old) were purchased from The Jackson Laboratory. Mice were maintained at 21°C on a 12-hour light/12-hour dark cycle; to help acclimate them to the new environment, they had free access to water and were fed a regular chow (Research Diets, D12492). Subsequently, mice were randomly separated into 2 exposure groups (*n* = 16/group). Mice were exposed through inhalation to either FA or PM_2.5_ (~10-fold over ambient level; ~60–120 μg/m^3^) for 6 hours/day, 5 days/week, for 24 weeks. Inhalation exposure was carried out in a Versatile Aerosol Concentrator and Exposure System (VACES) air pollution exposure facility at the Case Western Reserve University Animal Facility. The design of VACES has been described previously (14) and provides stable concentrations of PM_2.5_, which are roughly 10 times the ambient exposure. Biweekly exposure filters were collected and analyzed for the elemental concentration present in PM_2.5_ (Supplemental Figure 11).

### Glucose and insulin tolerance tests.

Glucose tolerance tests were performed after 14 weeks of PM_2.5_ exposure. Mice were fasted overnight for 16 hours; 2.0 g glucose/kg body weight was administered intraperitoneally. Blood glucose levels were measured in tail blood using a Contour blood glucose monitoring system at 0, 20, 40, 60, 90, and 120 minutes following glucose administration. Insulin tolerance tests were performed after 16 weeks of PM_2.5_ exposure. Mice were starved for 6 hours before intraperitoneal injection with insulin (Humulin R, Eli Lilly) at 0.75 IU/kg body weight. Blood was taken from the tail vein and glucose levels were measured using a Contour blood glucose monitoring system at 0, 20, 40, 60, 90, and 120 minutes after injection.

### In vivo indirect calorimetry.

Mice were housed individually for a total of 4 days in metabolic cages of the Promethion Metabolic Measurement System (Sable Systems International) during the 20 weeks of PM_2.5_ exposure. Only the 3 full light-dark cycles of data were used for analyses. VO_2_, VCO_2_, RER, energy expenditure (kcal), physical activity, and food and water intake were simultaneously measured for each mouse after a 1-day acclimatization period. RER, a measure of metabolism substrate choice (carbohydrate or fat), was calculated as the ratio between VCO_2_ and VO_2_. Energy expenditure indicates energy utilized during resting and activity period (nonshivering). All data collected were averaged over a monitoring period of 3 days.

### FDG uptake.

[^18^F]-fluorodeoxyglucose (FDG) was purchased from PETNET solution. After an 8-hour fast, mice were injected with insulin (0.75 U/kg) diluted in 0.9% physiological saline and 5 minutes later received an intravenous administration of FDG (200–300 μCi). After injection, the mice were maintained under conscious conditions and warmed using a heating pad. Before PET imaging, a computed tomography (CT) scout view was obtained to ensure mice were placed in the co-scan field of view (FOV) where the highest image resolution and sensitivity are achieved. Once the static acquisition was done, a CT acquisition scan was performed for attenuation correction. At 30 minutes, small-animal PET/CT (Inveon, Siemens) imaging were performed using an acquisition time of 15–30 minutes for PET at the Case Center for Imaging Research. Quantitative image analysis of the uptake of FDG in different organs was performed using Carimas II software (https://nmmitools.org/2019/01/01/carimas/). This program allows the regions of interest (ROIs) to be extrapolated from the reconstructed PET image frames, allowing the quantification of the SUV in a specific region. ROIs were defined based on the PET and 14 CT co-registered images from brain, liver, heart, muscle, WAT, and BAT and FDG tissue uptake was calculated using the mean of the SUVs.

### TEM.

BAT tissues were removed and immersed in 2.5% glutaraldehyde buffered to pH 7.4 with 0.1 M sodium phosphate and held for 2 hours for the first fixation. After rinsing with a sodium phosphate buffer, tissues were post fixed in 1% osmium tetroxide solution for 2 hours. Tissues were dehydrated in a series of graded ethanols, placed into propylene oxide, and embedded in Araldite. Tissues were cut with an ultramicrotome to ultrathin sections of 60–80 nm and stained with uranyl acetate and lead citrate and then examined under transmission electron microscope (JEOL 100 CXII) at 80–100 kV in the Cryo-Electron Microscopy core Laboratories at Cleveland Centre for Membrane and Structural Biology.

### ChIP-qPCR.

BAT tissue was isolated and flash frozen from FA- or PM_2.5_-exposed mice as indicated. Chromatin was isolated using the Imprint Chromatin Immunoprecipitation Kit (CHP1, Sigma-Aldrich) and performed per the manufacturer’s instructions. In brief, mouse BAT tissues were incubated with formaldehyde (1%, v/v) for 10 minutes at 37°C to crosslink the nuclear proteins to DNA. Subsequently, crosslinking was quenched by adding 1× glycine and incubation for 5 minutes at room temperature and BAT tissues were rinsed with ice-cold PBS and homogenized in lysis buffer containing protease inhibitor cocktail (homogenized with a Dounce homogenizer) followed by sonication and immunoprecipitation with IP-validated antibodies against HDAC9 (PA5-11245, Invitrogen), KDM2B (09-864, EMD Millipore), and H3K36me2 (ab176921, Abcam). IgG antibody and anti–RNA polymerase II antibody binding reactions were used as a negative and positive control, respectively. The captured chromatin was eluted and de-crosslinked, and the DNA was recovered. The ChIP-isolated DNA was subjected to PCR and qPCR analyses using primer pairs spanning the promoter region of *Nrf2*, *Rora*, *Gst1a*, and *Prdm16*, and the TSS of *Ucp1*. Fold enrichment was calculated by normalization to the average Ct value of input DNA and compared with *Gapdh*. ChIP-qPCR primer sequences are listed in Supplemental Table 10.

### In vitro mouse brown adipocyte culture and treatment.

Mature mouse brown adipocytes were obtained from the preadipocyte T37i cell line (Merck Millipore) by following the manufacturer’s recommendations. After differentiation, mature brown adipocytes were incubated with serum (10%) collected from mice exposed through inhalation to either FA or PM_2.5_ as described in *Experimental design and air pollution exposure* above. After 48 hours of incubation (in the presence or in the absence of *Hdac9* and *Kdm2b* gene depletion or overexpression), cells were harvested and used for bioenergetics study (oxygen consumption, glucose uptake, and lactate levels), assessment of ROS, and molecular biology analyses, including real-time PCR and mitochondrial functionality assessed by mitochondrial swelling assay.

### Hdac9 and Kdm2b knockdown and overexpression.

Transient transfection of mature brown adipocytes with commercially available mouse *Hdac9* and *Kdm2b* siRNA (Santa Cruz Biotechnology) was performed using Lipofectamine reagent (Invitrogen). A predesigned scrambled siRNA (Microsynth, 5′-UACACACUCUCGUCUCUdTdT-3′) was used as negative control. *Hdac9* and *Kdm2b* overexpression was achieved using *Hdac9* and *Kdm2b* CRISPR Activation Plasmids (Santa Cruz Biotechnology). Transient transfection was performed in antibiotic-free medium, and the medium was replaced after 24 hours. The following primer sequences were used for gene expression analysis: *Hdac9* forward, 5′-GAGCCCCAAATGAGGTTGGA-3′ and reverse, 5′-TGCCGTCACTTTGTACCCTC-3′; *Kdm2b* forward, 5′-TCTTTGAGTGCCGGGAGTTT-3′ and reverse, 5′-ACAAGTCCTCGTTCTCGTCG-3′; *Ucp1* forward, 5′-CCGAAACTGTACAGCGGTCT-3′ and reverse, 5′-TGATCCCATGCAGATGGCTC-3′.

### 3-Nitrotyrosine assay.

The levels of 3-nitrotyrosine, a marker of oxidative damage mediated by peroxynitrite, were measured in serum-treated mature brown adipocytes by a commercially available ELISA kit (ab116691, Abcam).

### Oxygen consumption rate and lactate level.

The oxygen consumption rate (OCR) and lactate levels in T37i cells were measured using the fluorescence-based Extracellular O_2_ Consumption Assay Kit (ab197243, Abcam) and the L-Lactate Assay Kit (ab65330, Abcam) respectively, following the manufacturer’s instructions. Briefly, cells were seeded in 96-well plates and recovered for 24 hours prior to the treatment with FA- and PM_2.5_-exposed mouse serum, either in the presence or absence of *Hdac9* and *Kdm2b* gene depletion or overexpression. OCR for each well was calculated and normalized to the cell number. Fluorescence intensities used to detect OCR are expressed as a percentage of FA serum. For lactate estimation, samples were subjected to deproteinization by the perchloric acid/KOH method, and fluorescence was measured at 587 nm.

### Glucose uptake.

Intracellular glucose uptake was measured using cell lysates with a Glucose Uptake Assay kit (ab136955, Abcam) according to the manufacturer’s instruction. Briefly, 2 × 10^3^ 3T3-L1 cells/well were seeded in 100 μL of serum-free DMEM/F12 overnight to increase glucose uptake. The following day, cells were starved for glucose by preincubating with 100 μL KRPH buffer containing 2% BSA for 40 minutes and then stimulated with insulin (1 μM for 20 minutes) to activate glucose transporter. Relative glucose uptake was determined using a standard curve.

### Mitochondrial swelling assay.

Mitochondria were isolated from T37i cells using a swelling buffer (250 mmol/L sucrose, 10 mmol/L MOPS, 5 μmol/L EGTA, 2 mmol/L MgCl_2_, 5 mmol/L KH_2_PO_4_, 5 mmol/L pyruvate, 5 mmol/L malate) and incubated with 150 μmol/L CaCl_2_ in a final volume of 200 μL in a 96-well plate for 20 minutes. Absorbance was read every 30 seconds at 520 nm.

### Statistics.

Results are presented as mean ± SEM. All data were tested for normal distribution and equal variance prior to use in parametric tests. Two-group comparisons were performed using Student’s 2-tailed *t* test. For multiple-group comparisons, 1-way and 2-way ANOVAs (repeated/nonrepeated measures where appropriate) were used to test for differences among the group means. Significant ANOVA interactions between variables were followed by Tukey’s multiple-comparison test. Tests of independence of categorical variables were carried out using χ^2^ tests. For all analyses, a *P* value of less than 0.05 was considered significant and false discovery rate (FDR) correction was used to account for type I error using the adjusted Benjamini-Hochberg method. For differential gene expression analysis, we used a cutoff of FDR less than 0.05.

### Study approval.

All animal experiments were approved by the Case Western Reserve University Institutional Animal Care and Use Committee (IACUC) and conducted according to institutional guidelines for ethical animal use (protocol 2016-0319).

### Data availability.

The data supporting the findings of this study are available in NCBI Gene Expression Omnibus (GEO) (transcriptome dataset: GSE145840; epigenome dataset: GSE255961). Values for all data points in graphs are reported in the Supporting Data Values file.

## Author contributions

SR designed the study and initiated PM_2.5_-based exposure design. RP performed FA/PM_2.5_ exposure as well as all in vivo experiments and conducted the FDG uptake, metabolic cage, gene expression, TEM, and histological studies. BP performed epigenomics and transcriptomic studies. JED and BP carried out all bioinformatics analyses and JED performed all statistical analyses. SC performed all in vitro studies and conducted the gene knockdown, gene overexpression, oxidative stress, bioenergetics, and mitochondrial functionality experiments. STM and EAC help to perform air pollution exposure. RP wrote the first draft of the manuscript. JED, BP, AVM, JEG, SB, FP, LCC, and MKJ contributed to revising the manuscript. SR edited and finalized the manuscript.

## Supplementary Material

Supplemental data

Supplemental data

Supporting data values

## Version 1 09/23/2025

Electronic publication
