# Supplementary material for: Air pollution modulates brown adipose tissue function through epigenetic regulation by HDAC9 and KDM2B
Source: JCI Insight. 2025 Sep 23;10(18):e187023. doi: 10.1172/jci.insight.187023 (PMC12548086; doi:10.1172/jci.insight.187023)
Supplement: Supplemental data [file jciinsight-10-187023-s151.pdf]

SUPPLEMENTAL MATERIALS

Air pollution modulates brown adipose tissue function through epigenetic regulation by HDAC9 and KDM2B

Rengasamy Palanivel[*1], Jean-Eudes Dazard*[1], Bongsoo Park[2], Sarah Costantino[3,4], Skanda T. Moorthy[1], Armando Vergara-Martel[1], Elaine Ann Cara[1], Jonnelle Edwards-Glenn[1], Shyam Biswal[5], Lung Chi Chen[6], Mukesh K. Jain[7], Francesco Paneni[3,4], Sanjay Rajagopalan[1,8].

[1] Cardiovascular Research Institute, Department of Medicine, Case Western Reserve University, Cleveland, OH, USA.

[2] Translational Gerontology Branch, National Institute on Aging, National Institute of Health, Baltimore, MD, USA.

[3] Center for Translational and Experimental Cardiology (CTEC), Department of Cardiology, University Hospital Zurich and University of Zurich, Schlieren, Switzerland

[4]University Heart Center, University Hospital Zurich, University of Zurich, Zurich, Switzerland.

[5] Department of Environmental Health and Engineering, Johns Hopkins University, Baltimore, MD, USA.

[6]Department of Environmental Medicine, New York University School of Medicine, New York, NY 100010, USA

[7] Division of Biology and Medicine, Brown University, Providence, RI, USA.

[8] Harrington Heart and Vascular Institute, University Hospitals, Cleveland, OH, USA.

[*]Denotes co-first authors

Supplemental Methods, Pages 2-9
Supplemental Figures (1-11), Pages 10-25
Supplemental Tables 9 and 10, Pages 26 -27
Reference, page 28

Supplemental Methods

Tissues collection for gene expression

In a separate exposure study, mice were exposed to FA vs $PM_{2.5}$(n=24/group) for 24 weeks, and brown adipose tissue (BAT) were collected at each Zeitgeber Time (ZT0 to ZT20) point (n=4) and we used n=3 or 4 at each ZT to analyze circadian variation in the expression of genes related to BAT differentiation, thermogenesis, substrate metabolism, and antioxidant defense.

Quantitative polymerase chain reaction.

Total RNA was extracted from the BAT and mature brown mouse adipocytes using TRIzol Reagent (Ambion, Thermo Fisher Scientific, MA) according to the manufacturer instruction. cDNA was synthesized using Transcriptor First Strand cDNA synthesis kit (Roche Applied Science, Indianapolis, IN) according to the manufacturer's protocol. The amplification of target genes was used by a LightCycler® 480 SYBR Green I Master kit (Roche Applied Science, Indianapolis, IN). Gene expression was measured by quantitative real-time PCR performed on a LightCycler® 480 real-time PCR System (Roche Applied Science, Indianapolis, IN). The sequences of real-time PCR primers used in this study are shown in Supplemental Table 10. Fold changes of mRNA levels were determined using the ΔΔCt method and normalized to internal control B2M or GAPDH.

Histology

The Brown Fat was harvested and immediately fixed in 10% formalin and embedded in paraffin. Paraffin sections were de-waxed for hematoxylin and eosin (H&E) staining, Picrosirius and Masson's trichrome staining. All images were captured under an inverted all in one fluorescence microscope-BZ-X710 (Keyence, Itasca, IL, USA).

Tissue collection and DNA/RNA extraction

BAT tissues were snap-frozen in liquid nitrogen and moved to -80°C until further processing. DNA/RNA was extracted from tissues using a QIAGEN All Prep kit after pulverizing the tissue using a Freezer Mill (6775 Freezer/Mill® Cryogenic Grinder). RNA quantity and quality were checked using NanoDrop (Thermo Fisher) and BioAnalyzer (Agilent Santa Clara, CA USA), respectively. Samples that had a RIN (RNA Integrity Number) value of 6.8 or higher were screened for this study.

Whole genome RNA-sequencing (RNA-seq)

The extracted RNA was quantified by NanoDrop (Thermo Fisher Scientific, MA) and the quality was assessed using a 2100 Bioanalyzer (Agilent Technologies, CA). The Agilent Bioanalyzer is a microfluidics platform used for sizing, quantification, and quality control for RNA (and DNA/ proteins) and provides an "RNA Integrity Number" (RIN), which quantifies the fragmentation of the RNA sample. The RNA samples were selected for sequencing if RIN value was more than 6.5. On average, 500-1,000mg of RNA samples were used for library preparation and double-stranded cDNA generation. We utilized TrueSeq RNA Library Prep Kit (Illumina, San Diego, CA) to generate strand-specific libraries for all other mentioned issues. The library was amplified by 15-cycles according to the manufacturer protocol. The prepared library was sequenced by HiSeq series sequencer including HiSeq4000, HiSeqX (Illumina, San Diego, CA). The raw BCL files were converted into FastQ files using CASAVA 1.8.2 (CASAVA). Raw sequencing reads were processed using the TaRGETII RNA-seq pipeline for large size RNA-seq sample processing (https://github.com/ShaopengLiu1/RNA-seq_QC_analysis). Prior to sequence reads alignment, we used trim galore (version 0.4.3) with cut adapt package (version 1.12) for sequence trimming and improving data quality.

Whole genome bisulfite sequencing (WGBS)

After attrition of mice samples (n = 3 / treatment group), genomic gDNA was extracted (500ng / sample) using QIAGEN All Prep kit. The quantity of DNA was by measured by picogreen method using victor X2 fluorometry (Lift technologies, cat#7589). The integrity of gDNA was completed by Agilent genomic DNA screen tape. The sample quality control criteria for WGBS library were above 7.0 of DIN score. Extracted gDNA was fragmented to average insertion size of 550 base-pairs, and the fragmented gDNA was converted to end-repaired adapters. The fragmented gDNA was bisulfite converted after size selection of EZ-96 DNA methylation Gold kit (Zymo, Irvine, CA USA) followed manufacturer's instruction. We applied Accel-NGS Methyl-Seq DNA library kit (Swift Biosciences, Catalog #30096). We have added dual indexing with Accel-NGS Methyl-Seq Unique Dual Indexing Kit (Swift Biosciences, Catalog #390384). Library quality control was performed using qPCR (LightCycler 480) and TapeStation 4200 (D1000 screen tape).

Whole genome DNA-accessibility sequencing (ATAC-seq)

All raw sequencing reads were trimmed using cutadapt package, and trimmed reads were mapped against mm10 genome using bowtie2 aligner. We used de-duplicated and uniquely mapped reads for peak calling analysis after excluding high-sensitive black-list regions defined by ENCODE. The candidate peaks were predicted by MACS peak calling software (FDR < 0.05). After identifying narrow peaks from FA and PM exposed BAT replicates, we created ~~a~~ merged consensus peaks and generated a matrix of open chromatin regions (OCRs). OCRs were subjected to quality control analysis to check for fragment size distribution and for detection of bad sample libraries showing sequencing depth / library size differences and possibly other distributional differences (Supplemental Figure 4A, Supplemental Figure 5, A-C).

Reference genome

Identifications of Differentially Methylated Regions (DMRs), Differentially Accessible Regions (DARs), and Differentially Expressed Genes (DEGs) as well as all subsequent analyses were done using the GENCODE M25 mouse reference assembly (GRCm39/mm39 - Ensembl release 108).

Identification of differentially methylated regions (DMRs)

We trimmed reads of adapter dimers using Trim Galore (0.4.3) and quality trimmed with minimum quality score above > 25. Attached adapter dimers were trimmed using a cut adapter tool. Firstly, bisulfite converted index (GA and CT conversion) were generated using the aforementioned mouse reference genome with Bismark build option, and trimmed reads were aligned with parameters bismark: bowtie2 -1 ($Trimmed read #1) -2 ($Trimmed read #2). Using the reads aligned to the lambda phage genome, library bisulfite conversion rate was estimated over 99%. Once we created aligned reads and corresponding locations, we used the bismark_ methylation_ extrator tool (bismark package) to summarize the level of methylation in CpG sites. A total of approximately 40-41 million sites per sample were predicted with DNA methylated sites (or unmethylated). After inspecting the potential bias to 5'-end regions, we applied exclusion of the first two base pair read regions: bismark_methylation_extrator -p –ignore 2 –ignore_r2 –comprehensive –no overlap –bedGraph –counts –buffer size 16G ($Aligned read bam file). Differentially Methylated Regions (DMRs) and blocks of differentially

methylated sites were identified with a minimum 5 CpG sites per block and at least > 5% Methylation difference between FA and $PM_{2.5}$ exposed-mice (t-test, p-value < 1e-4).

Identification of differentially accessible regions (DARs)

Gene-level raw counts of open chromatin regions/peaks OCR were calculated using the 'featureCounts' function of the Rsubread Bioconductor package. This resulted in a matrix of 40,838 candidate peaks for differential accessibility. These peaks were initially submitted to pre-filtering with associated genes, and those with a CPM mapped reads > 10 in all sample libraries and at least 15 mapped reads across all the sample libraries were kept. The resulting count matrix of 28,836 peaks was subjected to RUVr normalization to remove unwanted variations (Suppl. Fig.4b-4c). In this study, DARs were selected using a Bayesian Hierarchical Variable Selection method. We took advantage of the fact that the problem of differential accessibility regions can be cast into a variable selection problem in an ANOVA regression setting. This approach was proven to be very efficient to detect e.g. differentially expressed genes in high dimensional settings (1-3). We employed a method derived from such approach, called Bayesian ANOVA (BAM), a parameter estimation technique that relies on the so-called 'spike and slab' Bayesian hierarchical model used in model selection. This approach is far superior to conventional one-at-a-time (univariate) hypothesis testing procedures for several reasons. Unlike FDR controlling procedures, it will simultaneously control for false negative errors as well (1-3), eliminating the problem of specifying arbitrarily False Discovery Rate (FDR) cutoff values, and the drawback of excessive conservativeness (4). BAM also has optimal variable selection properties including variable selection consistency (in the statistical sense), meaning that with probability going to 1 BAM converges to the true model i.e. only true DARs are identified without false positives or negatives – as the sample size increases. In addition, Bayesian ANOVA makes minimal assumptions about the data are made. This makes it robust to non-normality and especially to correlations between variables, which is always present within large biological "omics" datasets. All calculations were performed in the R language using the implementation in an R package called 'SpikeSlab' available from the CRAN repository.

Identification of differentially expressed genes (DEGs)

Trimmed reads were mapped to the aforementioned mouse reference genome using STAR aligner, and gene-level raw counts were calculated using the 'featureCounts' function of the Rsubread Bioconductor package. For

differential gene/transcript expression analysis, the RNA-seq gene-by-sample matrix of read counts was analyzed using the DESEQ2 method. The differential expression analysis between FA and PM (or HFD) was performed after lane normalization, log-transformation, and removal of unwanted variation due to potential batch effect using RUVg. Differentially Expressed Genes (DEGs) were identified after applying the following cutoffs: FC > 1.2, CPM > 1.5, FDR < 0.05.

Gene annotations and final lists

All DMRs, DARs, and DEGs were annotated by chromosomic coordinates to get Ensembl 'Gene Location', 'Gene ID', 'Gene Symbol' and 'Entrez ID' using the aforementioned mouse reference assembly. Although the chromosomic coordinates are always informed, the 'Gene ID', 'Gene Symbol' and 'Entrez ID' gene annotations may be missing (NA), corresponding to intergenic regions of the genome. Further, gene annotations of DMRs, DARs, and DEGs are not necessarily unique due to possible matching of chromosomal coordinates to more than one gene. In this case all matches are included. This yielded three full lists of 881 annotated DMRs (464 hypomethylated and 417 hypermethylated - Supplemental Table 1a); 2278 annotated DARs (833 with a Gain of Accessibility (GA), and 1445 with a Loss of Accessibility (LA) - Supplemental Table 1b); and 678 annotated DEGs (409 up-regulated, and 269 down-regulated - Supplemental Table 1c). To ease the interpretability of the results and reduce the effect of noise on the error rates of our inferences during our integration analyses (see below), we further reduced the dimensionality of the data by selecting out the features with redundant or missing Gene IDs. This yielded three so-called curated lists of 441 unique fully annotated DMRs (234 hypomethylated and 237 hypermethylated - Supplemental Table 1a); 1861 unique fully annotated DARs (482 with a Gain of Accessibility (GA), and 1379 with a Loss of Accessibility (LA) - Supplemental Table 1b); and 663 unique fully annotated DEGs (402 up-regulated, and 261 down-regulated - Supplemental Table 1c). The same was done to keep unique and significant pairs (of significant regions and genes) after removing duplicated or missing gene annotations (Supplemental Tables 2A, 2B and Supplemental Table 4A, 4B).

Identification of DMRs and DARs target regulatory genes by GREAT or ATLAS analyses

Candidate open chromatin regions (genes, exons, introns, and UTRs) from the selected DMRs and DARs were submitted to search for potential transcription factor binding sites using HOMER software (5) using non-DMRs

and non-DARs as background. Identified DMRs less than 2kb from TSS were considered TSS-proximal sites, while DMRs beyond 2kb from TSS were considered TSS-distal sites. In addition, we performed differential motif binding analysis with available ChIP-seq datasets and summarized functional annotation of potential target genes using GREAT analysis. In this analysis, *de novo* motifs and known motif search were performed, and we reported top three to five *de novo* motif results. Finally, we also used the RGT-HINT software (6) to validate the identified transcription factors binding sites independently. These identified transcription factors binding sites were subjected to the same downstream analyses as above. Intersection of potential enhancer sites predicted by the Enhancer ATLAS database (7) and a Cell Metabolism study on BAT epigenome (H3K27ac/H3K4me1) (8) were used to identify DMRs and DARs target regulatory genes. To find regulatory sites for BAT from both resources, we downloaded BAT enhancer sites from the ATLAS database as well as raw FastQ files of H3K27ac and H3K4me1 ChIP-seq datasets and processed them to perform peak calling. We found that the Cell Metabolism study has more regulatory sites that overlapped with our DMRs and DARs. By using the intersection of the Enhancer ATLAS and BAT epigenome datasets (Suppl. Fig.6A) we aimed at detecting both robust and broad regulatory sites (including active and non-active enhancers).

Integrative analyses by multivariate partial least square modeling (MPLS*)*

Integration analysis of DMRs and DEGs results was done by a joint approach of correlation analysis and Multivariate Partial Least Square modeling (denoted MPLS). The multivariate version of PLS allows the modeling of a set of multivariate (p) independent variables from dataset X (n x p matrix) along with a set of multivariate (q) dependent variables from dataset Y (n x q matrix) on the set of common samples (n). MPLS finds a set of latent variables or components (h) in both matrices (X and Y) in such a way that their score values maximize their covariance (and correlation). Here, the MPLS model was tuned for the number of components by a minimum of percentage of cumulative explained variance for both the independent (X) and dependent variables (Y). Alternatively, this can be done by K-fold cross-validation if the sample size is large enough. MPLS assumes a direction of inference, that is, which dataset will be the multivariate dependent variables (Y) that we want to explain or predict by the other dataset of independent variables (X). Here, we alternatively set X: = DMRs (Differentially Methylated Regions) or X: = DARs (Differentially Accessible Regions), and Y: = DEGs

(Differentially Expressed Genes) after matching the common samples, that is, with $n = 6$, $p = 441$ (DMRs) or $p = 1861$ (DARs), and $q = 663$ (DEGs). For each set of (X, Y) datasets, we first consider all possible combinations of interaction pairs (p x q) between X and Y. We define so-called "homologous" pairs those interaction pairs for which GeneSymbol(X) = GeneSymbol(Y), and conversely "heterologous" pairs those interaction pairs for which GeneSymbol(X) ≠ GeneSymbol(Y). The fit of the MPLS model yields an estimated MPLS regression coefficient (β) for each possible DMRs-DEGs interaction pair: (p x q = 292,383, homologous: 21, heterologous: 292,362) and DARs-DEGs (p x q = 1,233,843, homologous: 73, heterologous: 1,233,770). Likewise, an estimated Spearman's correlation coefficient (ρ) was estimated for each possible combination (p x q) of DMRs-DEGs interaction pair. To assess the statistical significance of each DMRs-DEGs and DARs-DEGs interaction pairs, redundant pairs with identical pairs of 'Gene Symbols' were first removed. Second, those β and ρ coefficients only that passed a minimum percentile (in absolute value) of significance were retained: $|\beta| > \left|\beta^{(1-\alpha_\beta)}\right|$, where $\alpha_\beta = 0.01$ (99% percentile of regression coefficient β distribution) and $|\rho| > \left|\rho^{(1-\alpha_\rho)}\right|$, where $\alpha_\rho = 0.25$ (75% percentile of correlation coefficient ρ distribution). Finally, those interaction pairs only, that passed both criteria (heterologous) or either one criterion (homologous), that is, with a minimum absolute value regression coefficient and/or a minimum absolute value regression coefficient, were selected. The final selection criterion was chosen differently for the homologous and heterologous cases due to the mathematically very large imbalance that exists between their numbers in the first place. In our study, this analytical approach yielded 621 DMRs-DEGs significant interaction pairs (homologous: 5 - Supplemental Table 2A, heterologous: 616 - Supplemental Table 2B) and 4,255 DARs-DEGs significant interaction pairs (homologous: 13 - Supplemental Table 3A, heterologous: 4242 - Supplemental Table 3B). A two-component model ($h = 2$) was deemed sufficient for both the independent (X) and dependent set of variables (Y).

Functional annotations and analyses

For gene set enrichment analysis, we also utilized the relaxed cutoff (FDR < 0.05) to include more candidate DEGs with a minimum of five genes in the enriched gene sets. All Gene Ontology and KEGG pathway analyses were performed by Over Representation Analysis (ORA) (9) and Gene Set Enrichment Analyses (GSEA) (10),

two widely used approaches to determine whether known biological functions or processes are over-represented (enriched) in predefined sets of entities (GO terms, Gene Sets, Gene Pathways, etc.). Results and figures were generated with R packages 'TopGO', 'clusterProfiler', and 'enrichplot'. To validate our findings, Transfac, and HOMER analysis were performed to confirm upstream regulators from the DEGs list. Clustering analyses, heatmaps, and volcano plots were generated with R packages 'gplots' and 'RColorBrewer'.

Integrative analyses by TSS proximity

The given circadian and Nrf2 target genes were extracted from a public database. Circadian genes were extracted from Genecards/Pathway card database and Nrf2 target genes were extracted from Cistrom database and Hocomoco TF database (ver. 11). We considered TSS proximal sites (< 2kb) and additional distal sites (2-5kb) with observation from Nrf2 binding found in the Cistrome database (e.g., Hmox1). The negative or positive correlation was identified based on the TSS proximal sites only (+/- 2kb from TSS). When DNA methylation increases in TSS proximal sites along with an increase in the transcriptome genes, they are positively correlated (congruent) and vice-versa. Other correlation dataset may require additional validation using epigenetics makers (open chromatin, histone modifications, etc.).

Statistical analyses

Results are presented as means ± SEM. All data were tested for normal distribution and equal variance prior to use in parametric tests. Two group-comparisons were performed using Student's two-tailed t-test. For multiple-group comparisons, one-way and two-way ANOVAs (repeated/non-repeated measures where appropriate) were used to test for differences among the group means. Significant ANOVA interactions between variables were followed by Tukey's multiple comparison test. Tests of independence of categorical variables were carried out using Chi-square tests. For all analyses, a p-value < 0.05 was considered significant (*) and False discovery rate (FDR) correction was used to account for Type I error using the adjusted BH method. For differential gene expression analysis, we used a cutoff of FDR < 0.05.

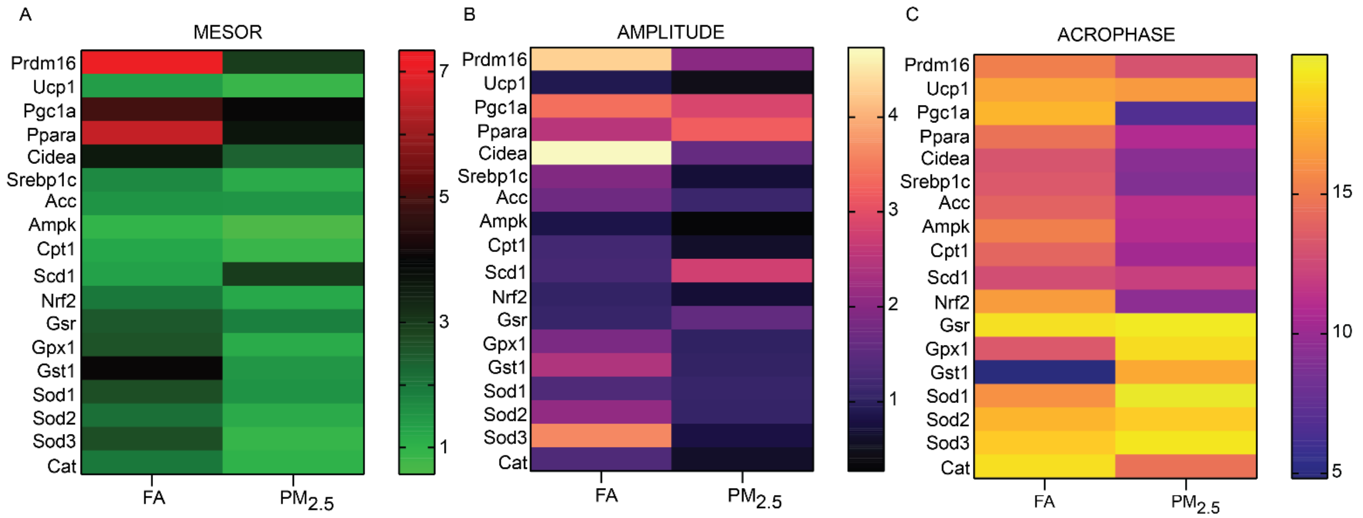

Supplemental Figure 1. Circadian rhythmicity measures of individual gene.

Cosinor Analysis of BAT Genes (Figure 1C). Heatmap indicate mean value of rhythmic parameters **(A)** MESOR, **(B)** amplitude and **(C)** acrophase. Differences in rhythmic parameters between FA vs $PM_{2.5}$ exposed mice revealed by unpaired Student t-test (n=3/group). Statistically significant differences: *$P < 0.05$ versus FA exposed mice.

Supplemental Figure 2

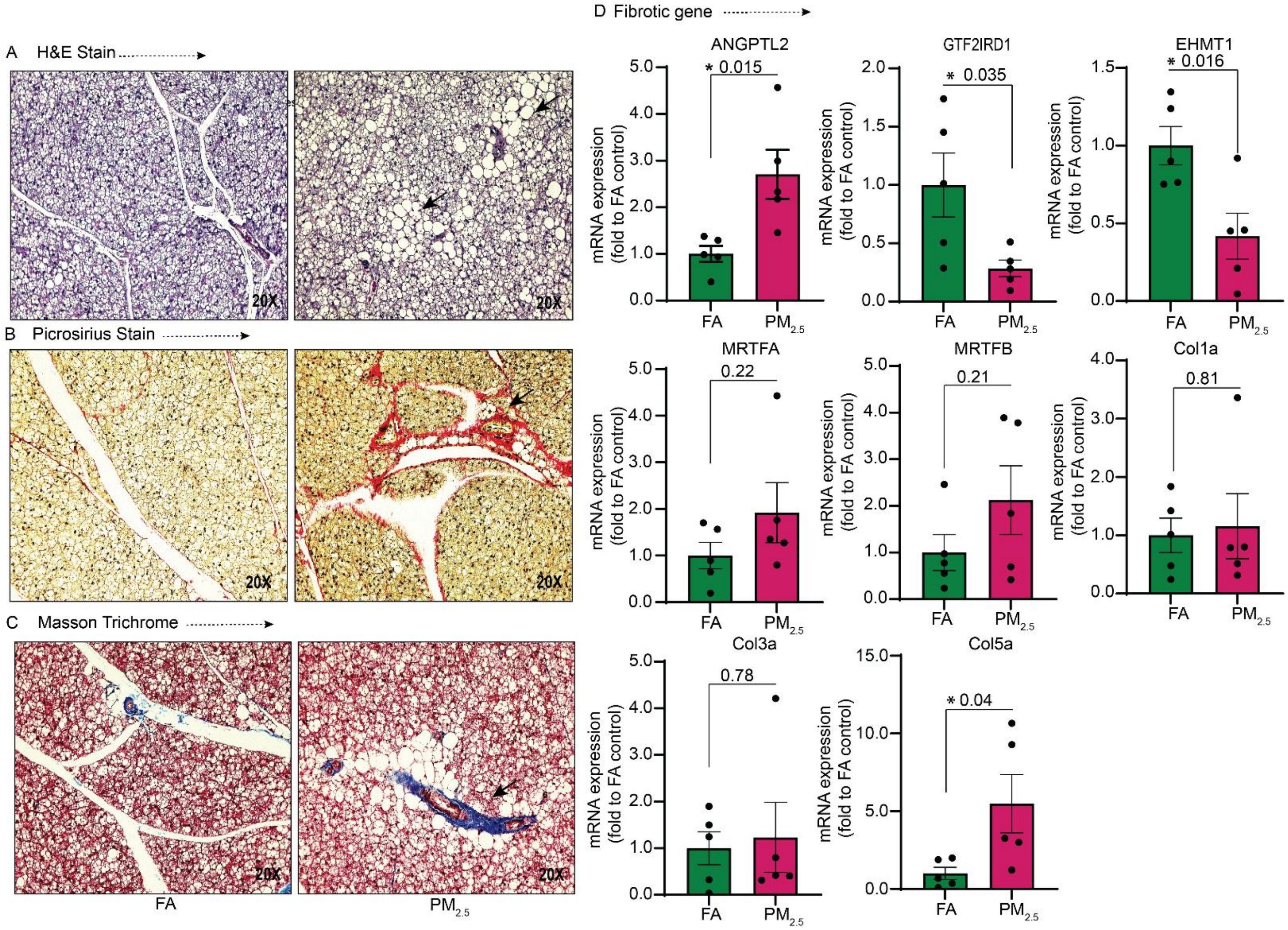

Supplemental Figure 2. Air particles ($PM_{2.5}$) induced fibrotic growth and associated genes in brown adipose tissue.

Representative photomicrographs of haematoxylin/eosin-stained BAT sections of FA vs $PM_{2.5}$ exposed mice. FA-BAT displays a normal morphological architecture (**A**). Representative photomicrographs of inflamed mice BAT tissues showing the distribution pattern of collagen fibers stained with Sirius Red (**B**), and Masson Trichrome (**C**), a red stain and blue stain indicate fibrotic accumulation respectively. (**D**) mRNA expression level of genes responsible for positive and negative implication of fibrotic growth in BAT from mice exposed with FA vs $PM_{2.5}$ for 24 weeks (n = 5/group). Data are provided as means ± SEM. Statistically significant differences: *$P < 0.05$ versus FA exposed mice (Unpaired two tailed Student's t test).

Supplemental Figure 3

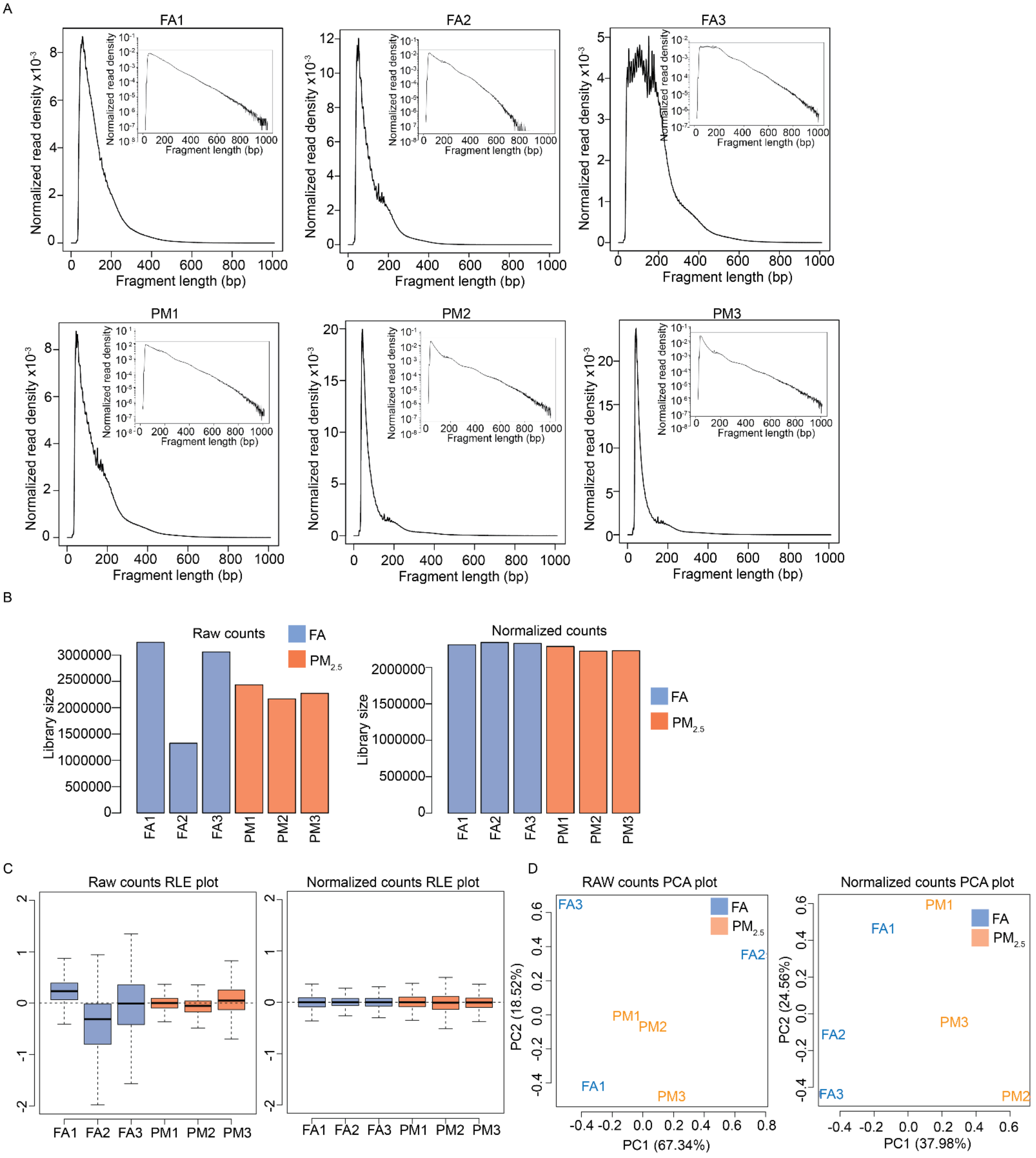

Supplemental Figure 3. ATAC-seq read counts quality control plots.

(**A**) Fragment size distribution by sample library. (**B**) Detection of bad sample libraries showing sequencing depth / library size differences and possibly other distributional differences: Barplots of total mapped read counts for

all sample libraries in raw data (left) and after normalization (right). (**C**) Removal of unwanted variation from the ATAC-Seq read counts: Relative Log Expression (RLE) plots. (**D**) Principal Component Analysis plots of unwanted variation from sample FA2 before (left) and after normalization (right).

Supplemental Figure 4

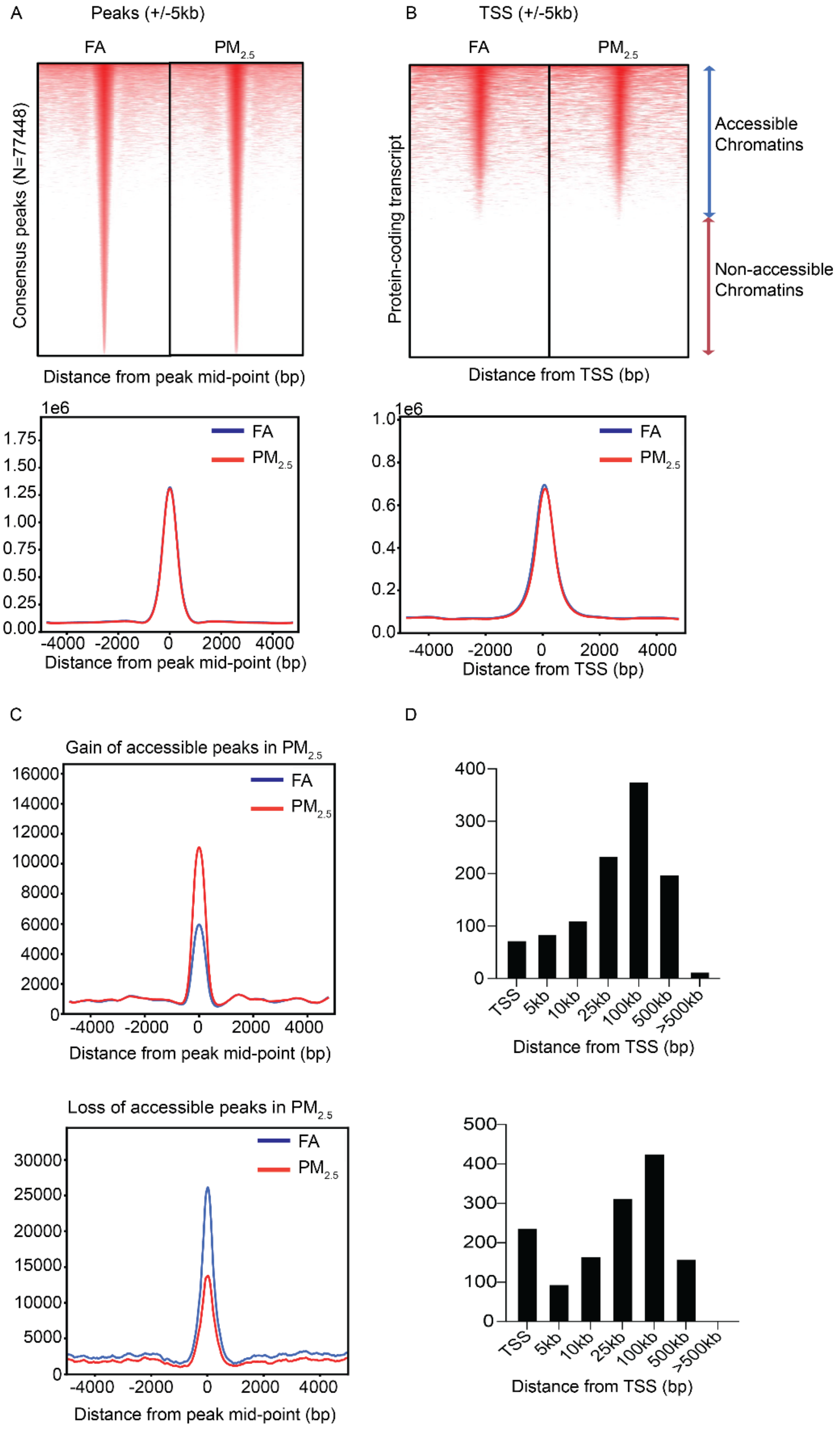

Supplemental Figure 4. ATAC-seq peak distributions comparisons.

(**A**) Heatmap (top) and composite plot (bottom) of ATAC-seq reads from the mid-point of peaks (+/- 5kb). (**B**) Heatmap (top) and composite plot (bottom) of ATAC-seq reads from the transcription start sites (+/-5kb). (**C**)

Gain of Accessible (GA) and Loss of Accessible (LA) peaks in PM2.5 exposed mice. (**D**) The histogram of the frequency of differentially accessible peaks grouped by the distance from TSS (bp).

Supplemental Figure 5

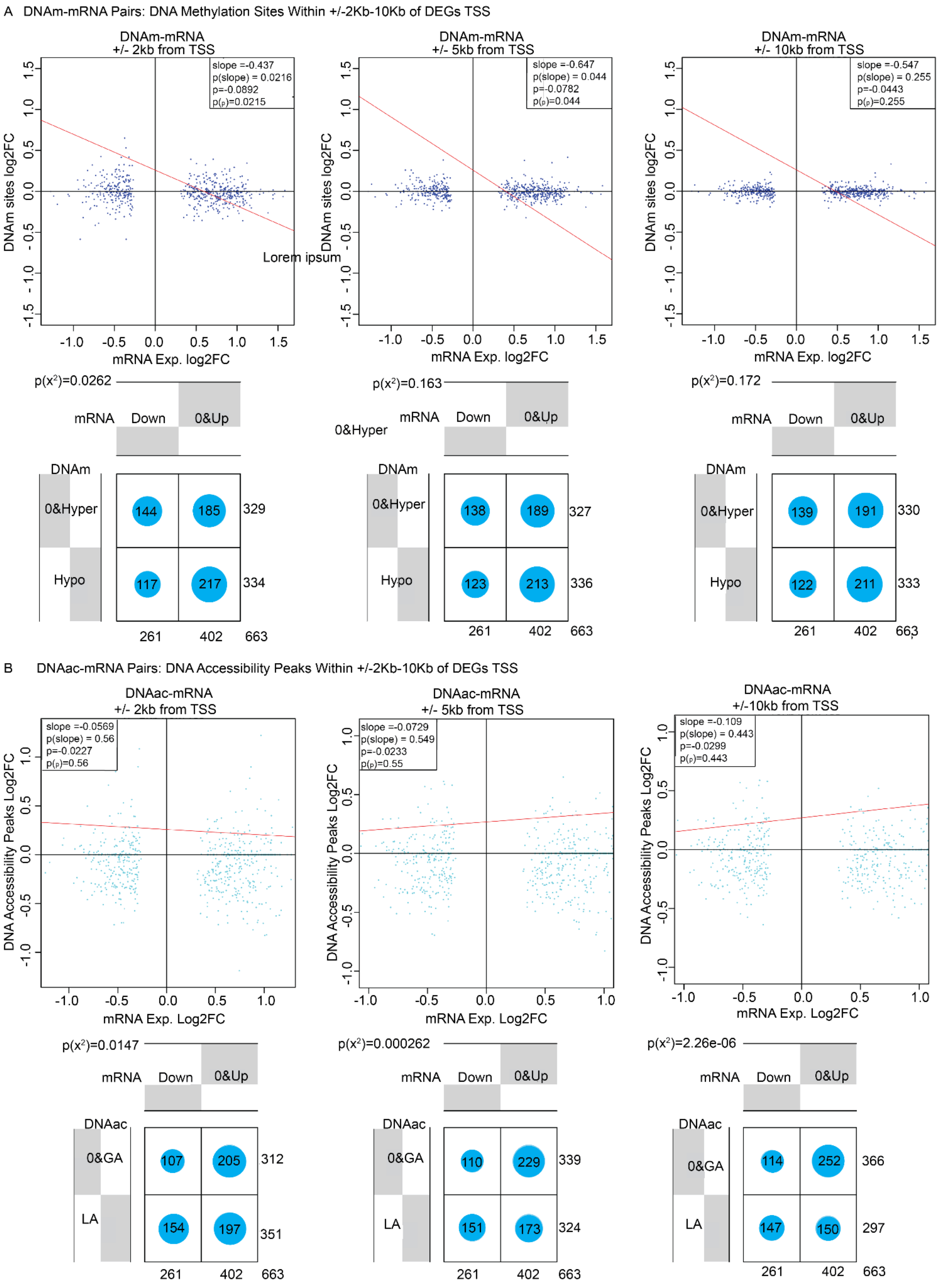

Supplemental Figure 5. Genomic region-level overlap analysis of DNA methylation and DNA accessibility vs. mRNA expression levels as a function of genomic distance from DEGs TSS.

Scatterplots of Trends of DNA Methylation and DNA Accessibility Levels vs. mRNA Expression Levels as a Function of Genomic Distance from DEGs TSS. Each point represents a pair of mRNA expression level of a DEG with its corresponding DNA methylation and/or DNA accessibility changes in its promoter and/or enhancer regions within +/-2Kb-10Kb of DEGs TSS. (**A**) DNAm-mRNA pairs: DNA methylation sites within +/-2Kb-10Kb of DEGs TSS. (**B**) DNAac-mRNA pairs: DNA accessibility peaks within +/-2Kb-10Kb of DEGs TSS. Results are shown for the significant DEGs (663). Corresponding log fold changes are used to plot each DNAm-mRNA and DNAac-mRNA pair. Top: Regression and correlation analyses showing regression slopes and correlation coefficients with their corresponding *p*-values. Bottom: categorical data analysis showing contingency tables with Chi-square tests of independence (or no association H_0) with their corresponding *p*-values. See also Figures 6 and 7.

Supplemental Figure 6

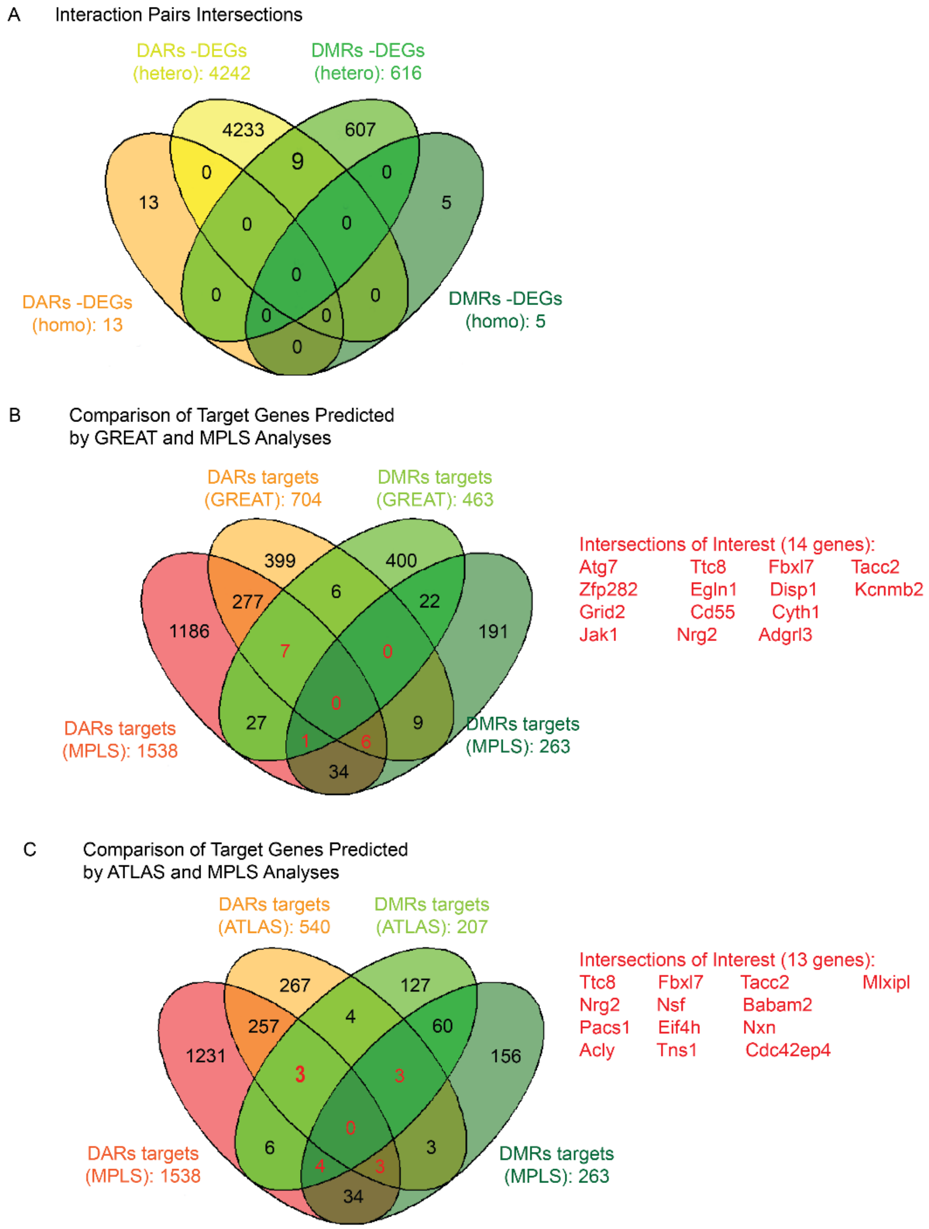

Supplemental Figure 6. Gene symbol-level intersection analyses.

(**A**) Venn-Diagrams of Gene Symbol-Level Intersections of Curated Genes and Regions and Their Interaction Pairs (see methods). 4-sets intersections between all DARs-DEGs and DMRs-DEGs curated interaction pairs identified by MPLS by interaction type (homologous or heterologous). Panels (**B**, **C**): Venn-Diagrams of Gene Symbol-Level Intersections of Target Genes Predicted by GREAT, ATLAS and MPLS analyses on Curated Interaction Pairs (see methods). (**B**) 4-sets intersection of DARs target genes identified by MPLS significant DARs-DEGs interaction pairs (red) with DARs target genes predicted by GREAT (orange). Intersection of DMRs target genes identified by MPLS significant DMRs-DEGs interaction pairs (dark green) with DMRs target genes predicted by GREAT (light green). Listed in red are 14 DARs and DMRs common target genes confirmed by at least two analytical methods (GREAT and MPLS) in at least one assay (DARs or DMRs). (**C**) 4-sets intersection of DARs target genes identified by MPLS significant DARs-DEGs interaction pairs (red) with DARs target genes predicted by ATLAS (orange). Intersection of DMRs target genes identified by MPLS significant DMRs-DEGs interaction pairs (dark green) with DMRs target genes predicted by ATLAS (light green). Potential enhancer sites from two epigenome data analysis studies were preselected for our ATLAS analysis (Supplemental Figure 6).

Listed in red are 13 DARs and DMRs common target genes confirmed by at least two analytical methods (ATLAS and MPLS) in at least one assay (DARs or DMRs). See also Supplemental Figure 6.

Supplemental Figure 7

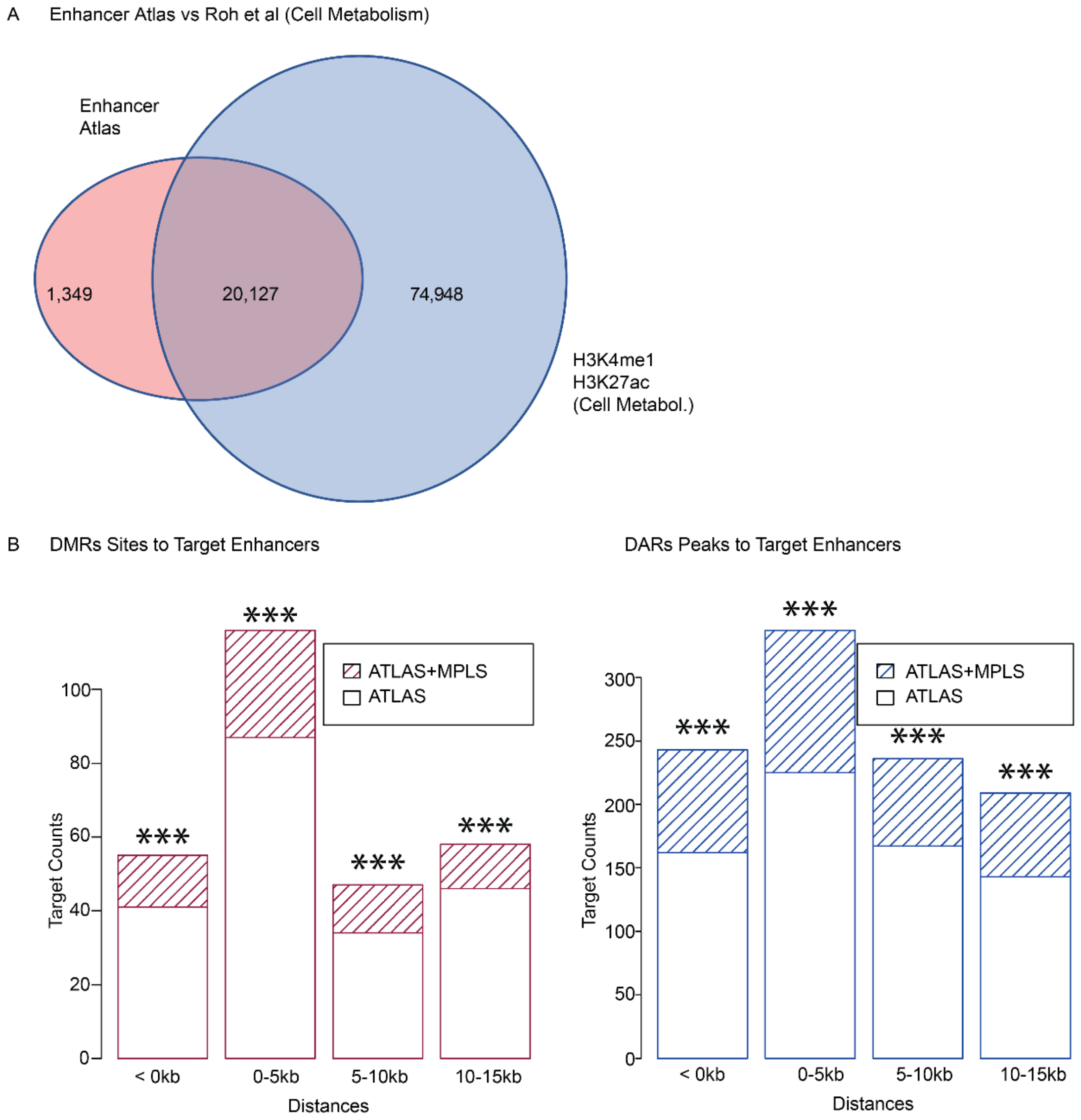

Supplemental Figure 7. Epigenome data analysis.

(**A**) Intersection of potential enhancer sites predicted by the Enhancer Atlas database (7) and a Cell Metabolism study (8). (**B**) Comparison between corresponding target genes predicted by GREAT and ATLAS analyses. The intersection of predicted enhancer sites from the above two epigenome data analytical studies was used for our ATLAS analysis (see methods). Left: Histogram of genomic distances distributions from DMRs sites to target enhancers; Right: Histogram of genomic distances distributions from DARs peaks to target enhancers. Statistical significance of overlap between ATLAS and MPLS predictions. Hypergeometric test *p*-values of significance are shown by bins.

Supplemental Figure 8

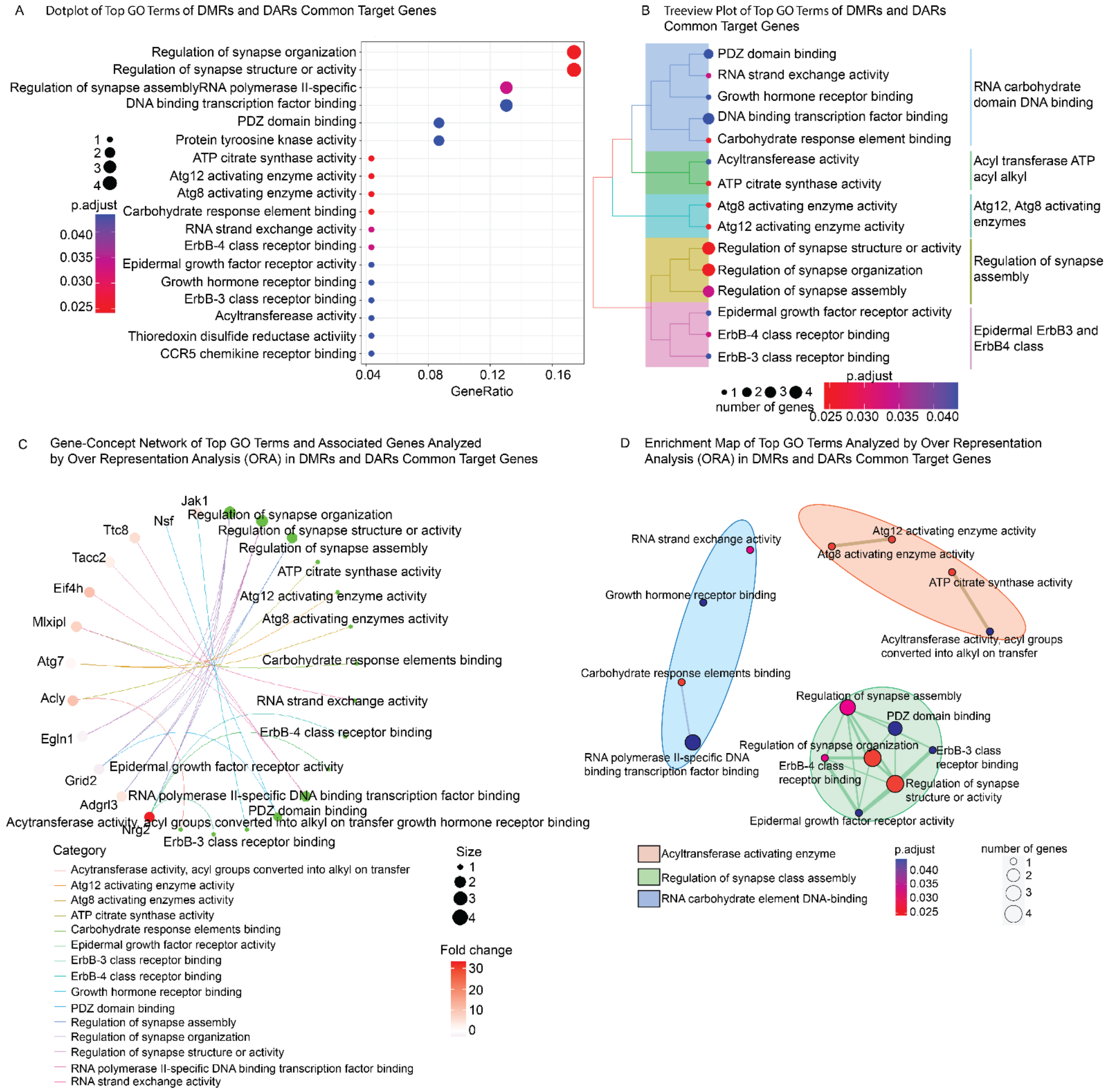

Supplemental Figure 8. Transcriptome data analysis of $PM_{2.5}$-exposed brown adipose tissue and integrative analysis with methylome data.

Gene Ontology (GO) of DMRs and DARs Common Targets Genes Analyzed by Over Representation Analysis (ORA). (**A**) Dotplot of significant (enriched) top regulated GO terms. (**B**) Treeplot view of significant (enriched) top regulated GO terms. (**C**) Gene-Concept Networks plot of significant (enriched) top regulated GO terms and associated genes. (**D**) Enrichment Map of significant (enriched) top regulated GO terms. The thickness of an edge between any two GO terms is proportional to the overlap between the two ontologies (see also Figure 6).

Supplemental Figure 9

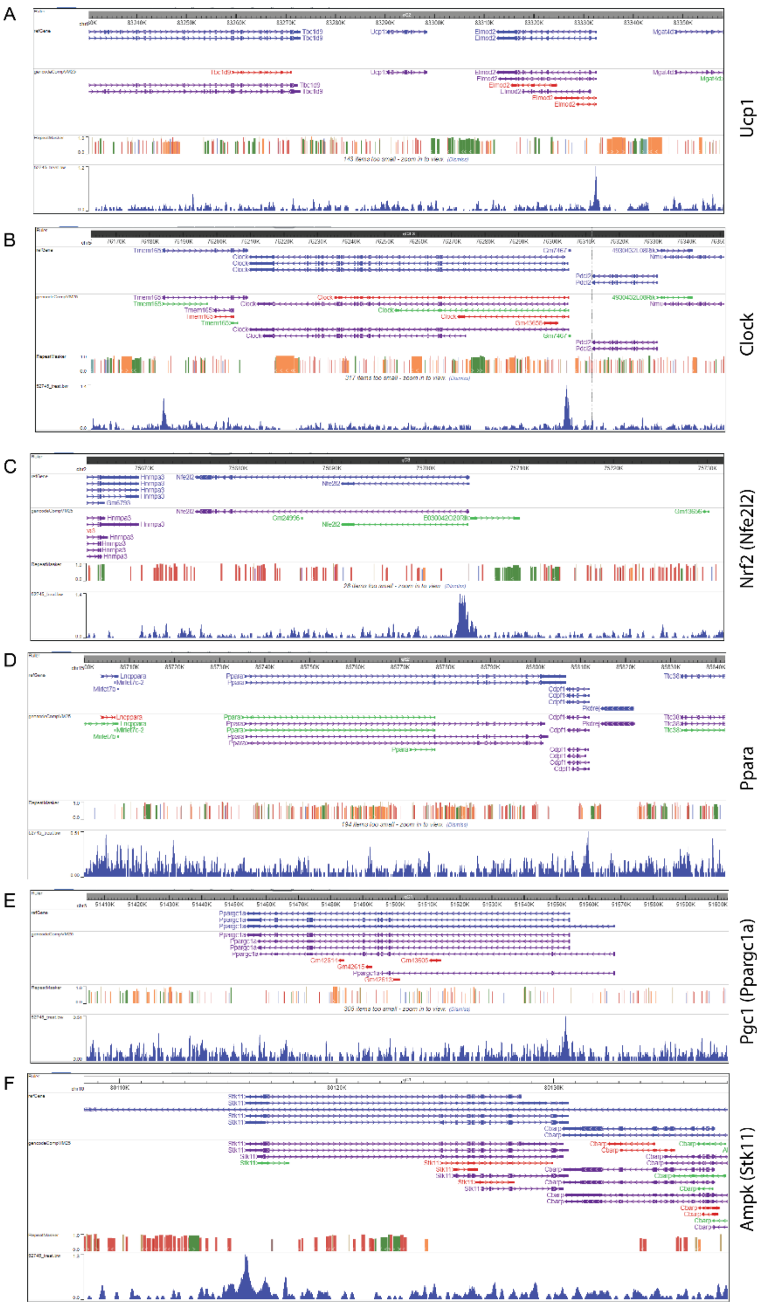

Supplemental Figure 09. Predicted *Kdm2b* target genes of interest.

Genome browser views of significant ChIP-seq *Kdm2b* predicted target binding in genes of interest involved in BAT Thermogenesis, BAT metabolism, Circadian Rhythm, and Redox Signaling. Results are from the Cistrome Database Browser, http://cistrome.org/db/#/ (11). (**A**) *Ucp1*, (**B**) *Clock*, (**C**) *Nrf2* (*Nfe2l2*), (**D**) *Ppara*, (**E**) *Pgc1* (*Ppargc1a*), (**F**) *Ampk* (*Stk11*).

Supplemental Figure 10

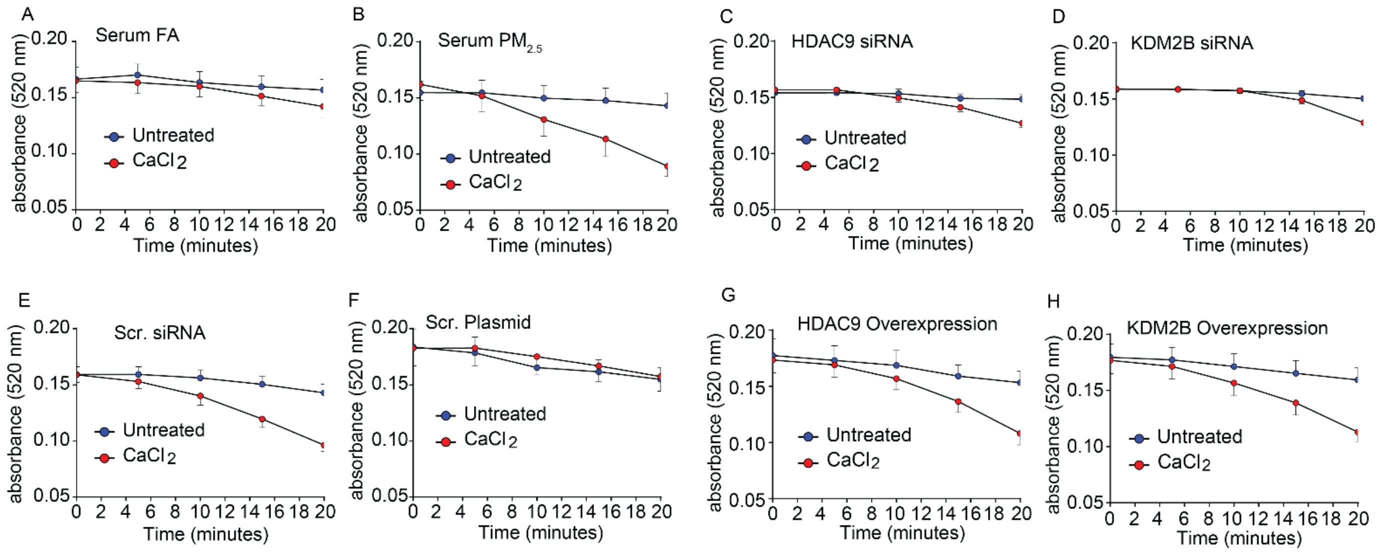

Supplemental Figure 10. Effect of siRNA-mediated knockdown and CRISPR-Cas9-mediated overexpression of Hdac9 and Kdm2b on $CaCl_2$ induced mitochondrial swelling. (**A**) Mitochondria isolated from BAT cell treated with serum from FA mice. (**B**) mitochondria isolated from BAT cell treated with serum from $PM_{2.5}$ mice. (**C-E**) mitochondria isolated from BAT cells transfected with either or Hdac9 or Kdm2b siRNAs or scrambled RNA (**F-H**) mitochondria isolated from BAT cells overexpressing either a scrambled plasmid or Hdac9 or Kdm2b (N=8/group). All mitochondria were treated with CaCl2 for 20 minutes. The data were obtained from three independent experiments.

Supplemental Figure 11

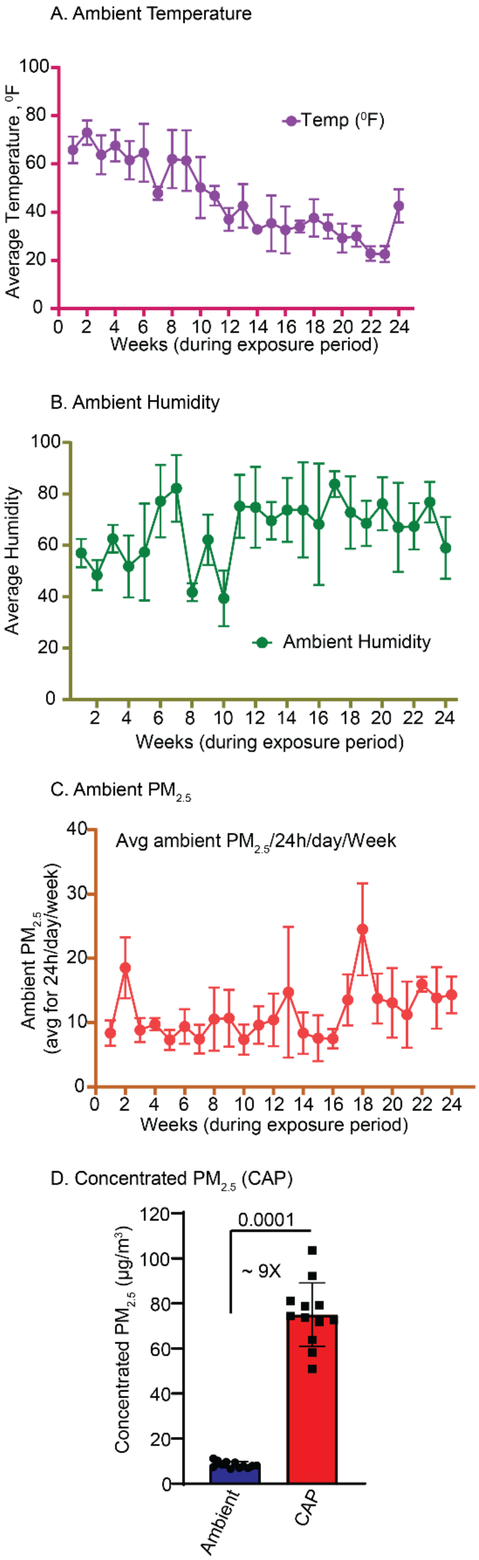

E. XRF Analysis- Chemical composition (Top 10 Elements)

Ambient Chemical Composition		Concentrated Chemical Composition	
Elements	Concentration(Mean±Std) (ng/cm^2)	Elements	Concentration(Mean±Std) (ng/cm^2)
S	824.86 ± 1048.39	S	2711.40 ± 1547.52
Fe	249.63 ± 290.66	Fe	1121.91 ± 618.21
Si	242.85 ± 312.54	Si	919.07 ± 579.93
Na	515.57 ± 204.74	Na	727.06 ± 466.00
Ca	198.78 ± 219.28	Ca	636.11 ± 440.09
Al	80.06 ± 81.45	Al	255.16 ± 144.97
K	81.14 ± 95.51	K	254.67 ± 147.23
Mg	112.50 ± 49.05	Mg	194.82 ± 55.17
Zn	102.00 ± 80.31	Zn	135.68 ± 55.14
U	95.44 ± 111.94	U	87.52 ± 143.06

F. XRF Analysis- Chemical composition (2nd Top 10 Elements)

Ambient Chemical Composition		Concentrated Chemical Composition	
Elements	Concentration(Mean±Std) (ng/cm^2)	Elements	Concentration(Mean±Std) (ng/cm^2)
Pr	13.95 ± 18.60	Pr	50.52 ± 43.60
Br	15.94 ± 21.11	Br	49.12 ± 29.00
Ti	12.78 ± 12.95	Ti	43.15 ± 23.17
Lu	17.90 ± 13.49	Lu	33.17 ± 12.57
Cu	13.68 ± 27.68	Cu	25.49 ± 10.11
Mn	8.49 ± 10.30	Mn	24.48 ± 12.05
Ni	6.17 ± 5.27	Ni	18.64 ± 12.37
Pb	5.69 ± 9.23	Pb	16.70 ± 9.35
Cr	3.90 ± 3.22	Cr	14.94 ± 6.42
Sb	10.10 ± 12.27	Sb	13.74 ± 2.49

Supplemental Figure 11. (**A**) indicate weekly average ambient temperature during the period of exposure (collected from www.timeanddate.com) (**B**) shows the weekly average ambient humidity during the period of exposure (collected from www.timeanddate.com) (**C**) depicts weekly average 24hr $PM_{2.5}$ level during the period of exposure (collected from www.epa.gov and www.airnow.gov) (**D**) shows the average ambient vs concentrated level of $PM_{2.5}$ level (weekly average for 12 bi-weekly exposure filter) during the exposure time.(**E, F**) describes

the elemental concentration contained in the ambient vs concentrated air. Data are provided as means ± Std. Statistically significant differences: *P < 0.0001 versus FA exposed mice (Unpaired two tailed Student's t test).

Supplemental Table 9. Summary of *Kdm2b* binding assay of target genes of interest.

ChIP-seq *Kdm2b* binding scores in target genes of interest involved in BAT Thermogenesis, BAT metabolism, Circadian Rhythm, and Redox Signaling. Results are from the Cistrome Database Browser, http://cistrome.org/db/#/ (11).

**Function**	**Gene**	**Score**	**Coordinate**
BAT Thermogenesis	Ucp1	**0.054**	chr8:83290347-83298455
	Prdm16	0	chr4:154316121-154358959
Circadian Rhythm	Bmal1 (Arntl)	0	chr7:113178470-113314125
	Clock	**0.986**	chr5:76209867-76304453
	Rora	0	chr9:68653403-68701774
Redox Signaling	Nrf2 (Nfe2l2)	**0.991**	chr2:75675512-75704662
	Gst1 (Chst1)	0	chr2:92599631-92615251
BAT Metabolism	Ppara	**0.013**	chr15:85734909-85801747
	Pgc1 (Ppargc1a)	**1.001**	chr5:51454248-52115852
	Ampk (Stk11)	**1.044**	chr10:80115794-80128562

Supplemental Table 10. Depicts the mouse primers list used in this study

Primers list	Forward primers	Reverse primers
Prdm16	CCACCAGCGAGGACTTCAC	GGAGGACTCTCGTAGCTCGAA
Ucp1	GGCATTCAGAGGCAAATCAGCT	CAATGAACACTGCCACACCTC
Pgc1a	AGCCGTGACCACTGACAACGAG	GCTGCATGGTTCTGAGTGCTAAG
Ppara	ACACTGCCAAGGAGTCGAG	AGGCATCTACCATGTCCATAA
Cidea	TGCTCTTCTGTATCGCCCAGT	GCCGTGTTAAGGAATCTGCTG
Srebp1c	CTGGAGCTGCGTGGTTT	GCCTCATGTAGGAATACCCTCCTCAT
Acc	GGATGACAGGCTTGCAGCTAT	TTTGTGCAACTAGGAACGTAAGTCG
Ampk	AAAGTGAAGGTGGGCAAGCA	CAGATGGTGTACTGATGACCTGG
Cpt1	CTGCTGCATGGTAGATGTTTCGAC	GCCCAGGAATGCTCTGCGTTTA
Scd1	CTCCTGCTGATGTGCTTCATCC	AGTGTATCGCAAGAAGGTGCTAAC
Nrf2	GATCCGCCAGCTACTCCCAGGTTG	CAGGGCAAGCGACTCATGGTCATC
Gsr	GCTATGCAACATTCGCAGATG	AGCGGTAAACTTTTTCCCATTG
Gpx1	GAAGAACTTGGGCCATTTGG	TCTCGCCTGGCTCCTGTTT
Gst1a	CGTCCACCTGCTGGAACTTC	GCCTTCAGCAGAGGGAAAGG
Sod1	GTCATTGGGATTGCGCAGTA	TGGTTTGACGGTAGCAGATGAGT
Sod2	TTAACGCGCAGATCATGCA	GGTGGCGTTGAGATTGTTCA
Sod3	CATGCAATCTGCAGGGTACAA	AGAACCAAGCCGGTGATCTG
Cat	TGAGAAGCCTAAGAACGCAATTC	CCCTTCGCAGCCATGTG
Gapdh	AACGACCCCTTCATTGAC	TCCACGACATACTCAGCAC
Fgf21	ACCTGGAGATCAGGGAGGAT	GTCCTCCAGCAGCAGTTCTC
Slit2	AACTTGTACTGCGACTGCCA	TCCTCATCACTGCAGACAAACT
Folistatin	TGGATTAGCCTATGAGGGAAAG	TGGAATCCCATAGGCATTTT
Endothelin1	TTCGTGACTTTCCAAGGAGC	GTCCATCAAGGAAGAACAGG
Angptl2	GGAGGTTGGACTGTCATCCAGAG	GCCTTGGTTCGTCAGCCAGTA
Nrg4	GAGACAAACAATACCAGAAC	GGACTGCCATAGAAATGA
Cxcl14	GGTCCAAGTGTAAGTGTTCC	CCTGGACATGCTCTTGGTG
Mrtfa	ACGAGGCGGTTACCATCAC	GCAGACAGAGACAGGAGCAC
Mrtfb	CGATAGCTCCAAGAAGCAGC	TTTTCTGGTTGCTTCCCTCA
Gtf2ird1	AAGAGAAAGAGGGTCTCTGAAGGC	CGTTTAGTCCGGAATAGTCCACCA
Ehmt1	GCTACATGGCCACCACAAA	TGCAGCTGCTAAAACAAAAGG
Sik2	TGAGCAGGTTCTTCGACTGAT	AGATCGCATCAGTCTCACGTT
ChIP-primers	Forward primers	Reverse primers
Prdm16	CCACCAGCGAGGACTTCAC	GGAGGACTCTCGTAGCTCGAA
Ucp1	TTTTGTTCTTGCACTCACGCC	CCATGGTGGGTTGCACTTC
Nrf2	GAGGTCACCACAACACGAAC	ATCTCATAAGGCCCCACCTC
Rora	CCCCTACTGTTCACCA	CCAGGTGGGATTTGGATATG
Gst1a	CGTCCACCTGCTGGAACTTC	GCCTTCAGCAGAGGGAAAGG

References

1. Ishwaran H, and Rao JS. Detecting differentially expressed genes in microarrays using Bayesian model selection. *J Am Stat Assoc.* 2003;98(462):438-55.
2. Ishwaran H, and Rao JS. Spike and slab gene selection for multigroup microarray data. *J Am Stat Assoc.* 2005;100(471):764-80.
3. Ishwaran H, and Rao JS. Spike and slab variable selection: Frequentist and Bayesian strategies. *Ann Stat.* 2005;33(2):730-73.
4. Genovese C, and Wasserman L. Operating characteristics and extensions of the false discovery rate procedure. *J Roy Stat Soc B.* 2002;64:499-517.
5. Heinz S, Benner C, Spann N, Bertolino E, Lin YC, Laslo P, et al. Simple combinations of lineage-determining transcription factors prime cis-regulatory elements required for macrophage and B cell identities. *Mol Cell.* 2010;38(4):576-89.
6. Li Z, Schulz MH, Look T, Begemann M, Zenke M, and Costa IG. Identification of transcription factor binding sites using ATAC-seq. *Genome Biol.* 2019;20(1):45.
7. Gao T, and Qian J. EnhancerAtlas 2.0: an updated resource with enhancer annotation in 586 tissue/cell types across nine species. *Nucleic Acids Res.* 2020;48(D1):D58-D64.
8. Roh HC, Tsai LTY, Shao M, Tenen D, Shen Y, Kumari M, et al. Warming Induces Significant Reprogramming of Beige, but Not Brown, Adipocyte Cellular Identity. *Cell Metab.* 2018;27(5):1121-37 e5.
9. Boyle EI, Weng S, Gollub J, Jin H, Botstein D, Cherry JM, et al. GO::TermFinder--open source software for accessing Gene Ontology information and finding significantly enriched Gene Ontology terms associated with a list of genes. *Bioinformatics.* 2004;20(18):3710-5.
10. Subramanian A, Tamayo P, Mootha VK, Mukherjee S, Ebert BL, Gillette MA, et al. Gene set enrichment analysis: a knowledge-based approach for interpreting genome-wide expression profiles. *Proc Natl Acad Sci U S A.* 2005;102(43):15545-50.
11. Inagaki T, Iwasaki S, Matsumura Y, Kawamura T, Tanaka T, Abe Y, et al. The FBXL10/KDM2B scaffolding protein associates with novel polycomb repressive complex-1 to regulate adipogenesis. *J Biol Chem.* 2015;290(7):4163-77.
